# YeeJ is an inverse autotransporter from *Escherichia coli* that binds to peptidoglycan and promotes biofilm formation

**DOI:** 10.1038/s41598-017-10902-0

**Published:** 2017-09-12

**Authors:** Marta Martinez-Gil, Kelvin G. K. Goh, Elze Rackaityte, Chizuko Sakamoto, Bianca Audrain, Danilo G. Moriel, Makrina Totsika, Jean-Marc Ghigo, Mark A. Schembri, Christophe Beloin

**Affiliations:** 1 Institut Pasteur, Unité de Génétique des Biofilms, 28 rue du Dr. Roux, 75724 Paris, CEDEX 15 France; 2 School of Chemistry and Molecular Biosciences, The University of Queensland, Brisbane, QLD 4072 Australia; 3 Australian Infectious Diseases Research Centre, The University of Queensland, Brisbane, QLD 4072 Australia; 4 Present Address: Departamento de Biología Celular, Genética y Fisiología, Facultad de Ciencias. Universidad de Málaga, Málaga, Spain; 5 Present Address: University of California San Francisco, Department of Medicine, San Francisco, CA USA; 6 Present Address: GSK Vaccines Institute for Global Health S.r.l., 53100 Siena, Italy; 7 Present Address: Institute of Health and Biomedical Innovation, and School of Biomedical Sciences, Queensland University of Technology, Kelvin Grove, QLD 4059 Australia

## Abstract

*Escherichia coli* is a commensal or pathogenic bacterium that can survive in diverse environments. Adhesion to surfaces is essential for *E. coli* colonization, and thus it is important to understand the molecular mechanisms that promote this process in different niches. Autotransporter proteins are a class of cell-surface factor used by *E. coli* for adherence. Here we characterized the regulation and function of YeeJ, a poorly studied but widespread representative from an emerging class of autotransporter proteins, the inverse autotransporters (IAT). We showed that the *yeeJ* gene is present in ~40% of 96 completely sequenced *E. coli* genomes and that YeeJ exists as two length variants, albeit with no detectable functional differences. We demonstrated that YeeJ promotes biofilm formation in different settings through exposition at the cell-surface. We also showed that YeeJ contains a LysM domain that interacts with peptidoglycan and thus assists its localization into the outer membrane. Additionally, we identified the Polynucleotide Phosphorylase PNPase as a repressor of *yeeJ* transcription. Overall, our work provides new insight into YeeJ as a member of the recently defined IAT class, and contributes to our understanding of how commensal and pathogenic *E. coli* colonise their environments.

## Introduction

*Escherichia coli* is a versatile bacterium comprising both commensal and pathogenic strains found in intra- and extra-intestinal environments^1^. The capacity for *E. coli* to adhere to different surfaces contributes to its ability to colonise and persist in specific host sites^2^. Among the numerous cell surface structures expressed by commensal or pathogenic *E. coli* multiple autotransporter (AT) proteins have been shown to contribute to their attachment and colonisation capacities^3–11^.

AT proteins represent a group of afimbrial adhesins that share several common features: an N-terminal domain leader sequence, a passenger domain that determines the functional characteristics of the protein and a C-terminal translocator or β-barrel domain that integrates into the outer membrane and facilitates transport of the passenger domain^12, 13^. In *E. coli* one of these autotransporters is the well-characterised phase-variable antigen 43 (Ag43) protein. Ag43 is encoded by the *flu* (or *agn43*) gene, and mediates cell-to-cell aggregation, biofilm formation, and long-term persistence in the urinary tract^3, 14, 15^. AT proteins have traditionally been grouped into four subclasses: Type Va, Vb, Vc and Vd^16^. However, more recent studies have revealed a new Type Ve subclass, which is similar to the classical monomeric Type Va AT proteins, but reversed in that they possess an N-terminal translocation domain and a C-terminal passenger domain^17, 18^. Due to this reversed topology, Type Ve AT proteins are also referred to as inverse AT (IAT) proteins. One such IAT is intimin, which mediates intimate binding to host cells and is an important virulence factor of enteropathogenic (EPEC) and enterohemorrhagic (EHEC) *E. coli* ^19^. The N-terminal region of some IATs contains a lysin motif domain (LysM domain), which mediates specific binding to peptidoglycan and acts as a dimerization interface for intimin^20, 21^.

Genomic analysis of the *E. coli* K-12 strain MG1655 revealed the presence of numerous genes encoding potentially cryptic adhesins, including AT proteins that contribute to attachment and colonization^9, 10^. In a previous study, we used the RExBAD cassette to place an arabinose inducible promoter upstream of putative adhesin-encoding genes in *E. coli* MG1655, and identified novel cryptic adhesins involved in biofilm formation that were not expressed under standard laboratory growth conditions^9^. This analysis identified YeeJ as an intimin-like protein involved in adhesion to different abiotic surfaces. While deletion of the *yeeJ* gene did not impact adherence to abiotic surfaces, the constitutive expression of YeeJ promoted strong biofilm formation^9^. The YeeJ protein from MG1655 is described as a 2,358 amino acid protein that belongs to the family of biofilm-associated proteins (Bap)^22^. Initially identified in *Staphylococcus aureus*, Bap is a surface adhesin that mediates biofilm formation and cell-to-cell adhesion, and can form amyloid fibers under specific environmental conditions^9, 23–25^. Bap-related proteins are present in many non-related Gram-negative and Gram-positive bacteria, including Esp from *Enterococcus faecalis*, LapA from *Pseudomonas fluorescens*, LapF from *Pseudomonas putida*, SiiE from *Salmonella enterica* and Bap from *Acinetobacter baumannii* ^26–30^. A common intriguing feature about these proteins is their very large size and ability to mediate a range of phenotypes, including (i) adhesion to abiotic and biotic surfaces, (ii) cell-to-cell interactions, (iii) biofilm formation, (iv) interaction with host epithelial cells and (v) the capacity to mediate invasion^22, 31^. Several studies have also shown these large extracellular adhesins also represent therapeutic targets, either as potential vaccines or as targets for anti-adhesion strategies^32, 33^.

In this study, we took advantage of the large number of *E. coli* genomes available on public databases to analyze the prevalence and conservation of the *yeeJ* gene. Our *in silico* analyses revealed the existence of two distinct variants of YeeJ that share similar functional properties. We show that YeeJ is both surface-located and present in the bacterial supernatant and that its LysM domain binds to peptidoglycan and is required for optimal YeeJ cell-surface localization and biofilm formation. At the regulatory level, our results revealed that, in *E. coli* K12, transcription of the *yeeJ* gene is increased in absence of the mRNA regulator PNPase. Taken together, this work enhances our understanding of YeeJ and its contribution to *E. coli* adhesion and biofilm formation.

## Material and Methods

### Bacterial strains and growth conditions

Strains used in this study are described in Table 1. Experiments were performed in LB medium or in M9 minimal medium with 0.4% glucose (M9-Glc) at 37 °C. Media were supplemented with the following antibiotics as required: ampicillin (100 μg/mL), chloramphenicol (25 μg/mL), kanamycin (50 μg/mL), spectinomycin (50 μg/mL), tetracycline (7.5 μg/mL) and zeocin (50 μg/mL).

**Table 1:** Strains and plasmids.

Strains	Description	Reference
MG1655	K-12 reference strain	*E. coli* genetic stock center
MG1655 Δ*yeeJ*	*yeeJ* gene replaced by a Zeo cassette, Zeo^R^	This study
MG1655 PcL*yeeJ*	*yeeJ* gene placed under the control of the *km*PcLrbs cassette λP_R_ promoter	This study
MG1655 PcL*yeeJ*ΔLysM	strain MG1655 PcL*yeeJ* lacking LysM domain using λred recombination	This study
MG1655 Δ*pnp*	Δ*pnp* moved by P1 vir from the KEIO collection mutant, Km^R^	This study
MG1655 Δ*pnp* (pZE12CFP)	pZE12CFP in MG1655, Amp^R^	This study
MG1655 Δ*pnp* (pPNP2)	pPNP2 in MG1655, Amp^R^	This study
UMN026	Wild type UPEC strain	87
MS427	MG1655*flu*	88
MS427 (pSU2718)	pSU2718 in MS427, Cm^R^	48
MS427 (pYeeJ_MG1655_)	pYeeJ_MG1655_ in MS427, Cm^R^	This study
MS427 (pYeeJ_UMN026_)	pYeeJ_UMN026_ in MS427, Cm^R^	This study
OS56	MG1655*flu*; GFP + ; Amp^R^	89
OS56 (pSU2718)	pSU2718 in OS56, Amp^R^ Cm^R^	48
OS56 (pYeeJ_MG1655_)	pYeeJ_MG1655_ in OS56, Amp^R^ Cm^R^	This study
OS56 (pYeeJ_UMN026_)	pYeeJ_UMN026_ in OS56, Amp^R^ Cm^R^	This study
BL21 (DE3)	F– ompT hsdSB(rB–, mB–) gal dcm (DE3)	Stratagene
MG1655 Δ*lacIZ::cat*	*lacIZ* operon replaced by a Cm cassette, Cm^R^	This study
MG1655 Δ*lacIZ* Δ*yeeJ*::*lacZ*	*yeeJ* replaced by the cassette *lacZ*zeo, RBS of the *yeeJ* gene conserved; ∆*lacIZ*::*cat*. Zeo^R^, Cm^R^	This study
MG1655_λATT::Km-GFPmut3	Source for cassette *km*PcL, Km^R^	49
Plasmids		
pSU2718	pACYC184-derived cloning plasmid	90
pYeeJ_MG1655_	*yeeJ* gene from MG1655 in pSU2718	This study
pYeeJ_UMN026_	*yeeJ* gene from UMN026 in pSU2718	This study
pZE12CFP	pZE12-MCS2-derived cloning plasmid	91
pPNP2	*pnp* gene from MG1655 in pZE12CFP	This study

### DNA manipulation and genetic techniques

Genomic DNA (gDNA) was extracted and purified using the Wizard Genomic DNA purification kit (Promega). Isolation of plasmid DNA was carried out using the QIAprep Spin Miniprep kit (Qiagen). Gel extraction and purification of PCR products were performed using the QIAquick Gel Extraction kit (Qiagen) and QIAquick PCR Purification kit (Qiagen) or MiniElute PCR Purification kit (Qiagen), respectively. PCR screening assays were performed with Taq polymerase (New England BioLabs), and PCRs requiring proofreading were performed with the Phusion^®^ High-Fidelity DNA Polymerase (New England BioLabs) or KAPA HiFi DNA Polymerase (Kapa Biosystems) as described by the manufacturers. Restriction endonucleases and T4 ligase were used per the manufacturer’s specifications (New England BioLabs). DNA sequencing was carried out using the BigDye^®^ Terminator v3.1 Cycle Sequencing Kit (Applied Biosystems) by the Australian Equine Genetics Research Centre.

### Bioinformatic analysis

The prevalence and sequence conservation of *yeeJ* was examined using the FASTA36 software package^34^ to probe 96 complete *E. coli* genomes on the National Centre for Biotechnology Information (NCBI) database (Figure S1). The prevalence of genes was determined using a cut-off of >75% over a 75% amino acid sequence alignment. The *E. coli* strains were classified into major phylogroups (A, B1, B2, D, E and F) based on an *in silico* analysis of the *arpA*, *chuA*, *yjaA* and TSPE4.C2 loci^35^. Amino acid alignments were performed using ClustalW^36^. The Conserved Domain Database (CDD)^37^, Phyre2^38^ and InterPro^39^ were used to analyze protein structures, and SignalP4.1^40^ was used to predict the presence of signal sequences. The genomic context of genes was analysed with Easyfig.^41^. The 118 strains collection is a subcollection of the 122-strains collection previously described^42^.

### PCR screening for the *yeeJ* gene in two *E. coli* strain collections

The prevalence of the *yeeJ* gene was assessed among strains from the ECOR collection^43^ and a subcollection of 118 strains from a previously described 122-strain collection^42^. Two primer sets were used: 3765 (5′-gatatgaacagcgagcaagc) and 3766 (5′-gtcattttcgccctgttta) targeting a 669 bp fragment at the 5′ end of the gene, and 3767 (5′-cagaagacaaaataatgagcgg) and 3768 (5′-ggtttttataacatgtcgcataagc) targeting a 591 bp fragment at the 3′ end of the gene. Primers 4225 (5′-gatcaaagtactgctgccctg) and 4226 (5′-gctatcgacgccattacctg) were used to further screen for the 906 bp fragment. Genomic DNA extracted from strains from the two collections was used as template DNA in the PCR assays.

### β-galactosidase assays

β-galactosidase activity was measured as described previously^44^. Overnight cultures were washed and concentrated twice in LB medium. The enzyme activity was measured in triplicate for each strain at 37 °C with two technical repeats per sample. Colour development was measured at an optical density of 420 nm (OD_420_) in 1-cm cuvettes. Data were presented in arbitrary Miller units and calculated using the following formula: (750 * OD_420_)/(T * V * OD_600_), where T is time in minutes and V is volume in ml.

### Biofilm formation assays

PVC 9-well microtiter plates (BD Falcon) were used to monitor biofilm formation as described previously^45^. Briefly, M9-Glc minimum media containing 1 mM IPTG was inoculated with a 1/100 dilution from an overnight culture in M9-Glc minimum media. After inoculation, microtitre plates were incubated at 37 °C for 24 h, rinsed and 150 μl of a 0.1% solution of crystal violet was added to each well. The plates were incubated at room temperature for 30 min and rinsed, and biofilm formation was tested as follows: crystal violet was solubilized by addition of 150 μl of ethanol-acetone (80:20), and the OD_595_ was determined. Results were presented as the mean of four replicate wells in three independent experiments. Flow cell assays were performed as previously described^46^. Briefly, OD_600_ standardized cells pre-grown in M9-Glc minimal media containing the appropriate antibiotics and 1 mM IPTG, if required, were inoculated into flow chambers and biofilms were allowed to develop on plastic coverslips (ProSciTech, Kirwan, QLD, Australia). Scanning confocal laser microscopy was performed using a ZEISS LSM 510 META Confocal Microscope to monitor biofilm formation 24 h post-inoculation.

### Immunofluorescence microscopy

Overnight cultures supplemented with the appropriate antibiotics and 1 mM of IPTG were fixed to an OD_600_ of 0.4, spotted onto a glass slide and allowed to dry. The cells were fixed with 4% paraformaldehyde (PFA) and quenched with 50 mM NH_4_Cl. After three washes with PBS, the slides were blocked with 0.5% BSA, and incubated with a 1:100 dilution of the appropriate primary antibody in PBS for 30 minutes. The cells were washed with PBS and incubated with a secondary goat anti-rabbit antiserum coupled to fluorescein isothiocyanate (FITC), diluted 1:500 in PBS. The slides were washed and air-dried, mounted with ProLong Gold (Invitrogen), and examined under a ZEISS Axioplan 2 epifluorescence microscope.

### Protein preparation from outer membrane vesicles (OMVs)

Overnight cultures were diluted 1/100 into 50 ml of LB broth with the appropriate antibiotics and induced with IPTG as required. After four hours of growth, cells were fixed at an OD_600_ of 1.0 and pelleted at 4 °C. Cell pellets were washed three times with 1 ml of cold PBS, and resuspended in 1 M of ethylenediaminetetraacetic acid (EDTA) buffer. Samples were incubated at 56 °C for 30 min and centrifuged for 15 min at 4 °C. The supernatants were filter sterilized using 0.22-μm filters. Trichloroacetic acid (TCA) was added to the samples to a final concentration of 20% to precipitate proteins. After an overnight incubation at 4 °C, cold acetone was added to each sample and the samples were centrifuged for 1 h. This wash was repeated twice. Pellets were air-dried and resuspended in 50 μl of resuspension buffer (50 mM ammonium bicarbonate, 3 M urea, 5 mM DTT). The samples were mixed with 50 μl of 2xSDS loading buffer, boiled for 5 min and centrifuged. Commercial anti-OmpA antibodies were used as a control (Antibody Research Corporation).

### Peptidoglycan binding assays

Peptidoglycan (PG) from *E. coli* (Invitrogen) was used for pull-down assays. One milligram of lyophylized peptidoglycan was resuspended in 1 ml of 50 mM Tris-HCl, pH 7, sonicated (Misonix Sonicator 2000; microprobe, 30 s) and used as a stock for pull-down assays as described^47^. Cell pellets were lysed by B-Per and the protein fraction from the soluble phase was recovered. Protein extracts were concentrated 100× by size exclusion centrifugal filtration and dialyzed overnight against 50 mM Tris-HCl, pH 7. 100 μg of total protein extracts were incubated with 150 μl of the 1 mg/ml peptidoglycan stock (0.15 mg PG final concentration) in a total reaction of 300 μl. A control reaction lacking peptidoglycan was included. Reaction mixtures were incubated for 2 h at 4 °C with gentle agitation and then centrifuged at 20 000 × *g*. The supernatant containing unbound protein (U) was removed and mixed with SDS sample buffer for Western blot analysis. The pellet containing peptidoglycan and bound protein (B) was treated with 300 μl of 4% SDS in 50 mM Tris-HCl, pH 7 for 15 min at 4 °C and centrifuged. The supernatant containing released protein was collected and mixed with SDS sample buffer. Samples were analysed by Western immunoblotting using anti-YeeJ antibody raised against the C-terminal domain.

### SDS-PAGE and Western blot analysis

Cell lysates, OMVs extractions and bacterial concentrated supernatants were subjected to SDS-PAGE and were transferred to PVDF microporous membrane filters as previously described^48^. Culture supernatants were prepared by filtering (pore size 0.22 μm) to remove intact bacterial cells and concentrated 100× by size exclusion centrifugal filtration. Serum raised against different domains of YeeJ (β-barrel and C-terminal domain) was used as primary antibody. The secondary antibody was an alkaline phosphatase-conjugated anti-rabbit immunoglobulin G. 5-Bromo-4-chloro-3-indolylphosphate (BCIP)-nitroblue tetrazolium (NBT) was used as the substrate in the detection process (Healthcare).

### Construction of strains and transposon mutagenesis

The strain constitutively expressing the YeeJ protein from *E. coli* MG1655 was constructed by the insertion of the λP_R_ constitutive promoter upstream of the *yeeJ* coding region to generate MG1655 PcL*yeeJ*. The strain constitutively expressing a YeeJ protein deleted for the LysM domain was constructed using lambda-red recombination of a PCR fragment constructed by overlapping PCRs to generate MG1655 PcL*yeeJ∆*LysM. Strain MG1655 PcL*yeeJ* was used as a template for PCRs using primers: yeeJ.500-5 (5′-gttcattaattaacaactttg), delta-LysM-3 (5′-tttttttcactaacttgtgccaccgtattggcatttgcaatggca) and delta-LysM-5 (5′-ttgcaaatgccaatacggtggcacaagttagtgaaaaaaaattaa) and LysM-500-3 (5′-caggtgcgctgcgccagttggt). The two fragments obtained were assembled by PCR using yeeJ.500-5 and LysM-500-3. The assembled fragment was used to recombine using lambda red on the MG1655 genome and selection was performed using kanamycin. The insertion of the PcL promoter and the LysM deletion were verified by PCR with LysM-ext-3 (5′-tggccgggcgtgcttcataatc) and LysMjunction-5 (5′-ccaatacggtggcacaagttag) and sequencing using LysM-seq (5′-gacaaatattatgcgggatg). The MG1655∆*yeeJ*::*lacZ* strain was constructed using lambda red recombination employing the following primers: yeeJ-lacZzeo-Long.L-5 (5′-aagtggagaagaaataaatgaccgacaaatattatgcgggatgaccatgattacggattcactg), yeeJ-lacZzeo-Long.L-3 (5′-gccccgaatggaacactctttaagaaattataacggaaaatcagtctcctgctacgaa). Verification was performed using primers YeeJ.ext-5 (5′-gcatcacgctaatacagactcg), YeeJ.ext-3 (5′-gtctaccgtttatccttaccac), lacZ.ATG + 100-3 (5′-gggggatgtgctgcattaag) and zeo.verif-5 (5′-caggaccaggtggtgccggacaacaccc). The MG1655Δ*pnp* strain was constructed using P1 transduction to transfer the *pnp* mutation from the KEIO collection to strain MG1655 using primers PnpFWDmut (5′-gtgccgtaaggtactgtctaag) and PnpREVmut (5′-gcagcgggagggcaatggc). All constructs were finally verified by sequencing. Random mutagenesis using *mariner* transposon was performed on strain MG1655Δ*yeeJ*::*lacZ*Δ*lacZI* as described previously^49^. Transposon insertion sites were determined using arbitrary PCR. Homology searches were performed using Blast 2.0.

### Construction of plasmids

The *yeeJ* gene was amplified from either *E. coli* K-12 strain MG1655 or UPEC UMN026 with primers 3797 (5′-ccggcgtcgacatgaaggagggtaagcatggctacgaagaagag) and 3764 (5′-ggccggcatgctcagaggtttttataacatgtcg). The PCR products were digested with SalI (forward primer) and SphI (reverse primer), and ligated to a SalI-SphI digested pSU2718 plasmid to generate pYeeJ_MG1655_ and pYeeJ_UMN026_, respectively. Transcription of *yeeJ* in these constructs was under the control of the IPTG-inducible *lac* promoter. The *pnp* gene was amplified from K-12 strain MG1655 with primers PnpFWD (5′-ggtaccttgcttaatccgatcgttcg) and PnpREV (5′-aagcttttactcgccctgttcagcagc). The PCR products were digested with KpnI (forward primer) and HindIII (reverse primer) and ligated to a KpnI-HindIII digested pZE12CFP plasmid to generate pPNP2 plasmid.

### Detection of YeeJ by ELISA

100 µl of bacterial culture adjusted to OD_600_ = 1 in a carbonate coating buffer (0.1 M NaHCO3, 0.1 M Na2CO3; pH to 9.5) were inoculated into 96 well microtiter plate MaxiSorp (Nunc.) and incubated overnight at 4 °C. Bacterial culture was removed by inversion, washed with T-PBS (×3) and incubated with 2% Milk/T-PBS for 1 hour at room temperature. Primary antibodies were diluted 1/100 for anti-YeeJ and 1/1000 for anti-*E. coli* in 1% Milk/T-PBS and incubated 1 hour at RT and washed with T-PBS (×3). Secondary antibody anti-rabbit IgG HRP was diluted 1/10000 in 1% Milk/T-PBS, incubated 1 hour at RT and washed with T-PBS (×3). Substrate reagent R&D Systems DY999 and Stop solution R&D Systems DY994 were used to develop and absorbance was read at OD_420_. The results are averages for four replicate wells in three independent experiments.

### RNA extraction and qRT-PCR

500 µl of exponentially growing cells (OD_600_ = 0.6) were stabilized in 1 ml of RNAprotect Bacteria Reagent (Qiagen), and subsequent RNA extraction was performed using the RNeasy Mini Kit (Qiagen). After treatment with rDNase I (Ambion) to remove contaminating gDNA, the RNA was re-purified using the RNeasy Mini Kit RNA cleanup protocol (Qiagen). First-strand cDNA synthesis was performed using the SuperScript® III First-Strand Synthesis System (Invitrogen) as per manufacturer’s recommendation. Real-time PCR was performed using SYBR® Green PCR Master Mix (Applied Biosystems) on the ViiA^TM^ 7 Real-Time PCR System (Applied Biosystems) using primers 6965 (5′-tttgcgactgaccaactg) and 6966 (5′-agaccagtttaccgccaa) for *yeeJ*. Transcript levels of each gene were normalized to *gapA* as the endogenous gene control using primers 820 (5′-ggtgcgaagaaagtggttatgac) and 821 (5′-ggccagcatatttgtcgaagttag). Gene expression levels were determined with the 2^−ΔΔCT^ method, with relative fold-difference expressed against wild type MG1655. All experiments were performed as three independent replicates.

### Purification of YeeJ and generation of antibodies

Two six-histidine tagged, truncated forms of YeeJ were constructed. Primers 3096 (5′-tacttccaatccaatgcgaacctcgagcaacagatagcc) and 3097 (5′- ttatccacttccaatgtcacgttgtgaccactttaccacc) were used to amplify the portion of the gene encoding for the predicted β-barrel domain of the protein (corresponding to amino acids 119–460), and primers 5560 (5′-tacttccaatccaatgcggatgaaaaactgacactcac) and 5561 (5′-ttatccacttccaatgtcagcttgagttgccagtga) were used to amplify a portion of the predicted extracellular passenger domain of the protein (corresponding to amino acids 1838–1938). The PCR products were purified and inserted into the pMCSG7 vector by ligation independent cloning. The plasmids were transformed separately into *E. coli* BL21(DE3), and expression of the recombinant proteins was induced with 1 mM IPTG. The recombinant proteins were purified by Ni-nitrilotriacetic acid (NTA) superflow columns (Qiagen) under denaturing conditions (according to manufacturer’s instructions). The purified proteins were quantified with the Bicinchoninic Acid Protein Assay Kit (Sigma) and assessed for purity via SDS-PAGE. Rabbit polyclonal antisera were raised against each recombinant protein using four immunizations (400 µg protein/dose) at the Walter and Eliza Hall Institute Antibody Facility. The antisera were adsorbed against a crude protein extract of MG1655 Δ*yeeJ* prior to use.

## Results

### Prevalence and conservation of the *yeeJ* gene

**Figure 1:**
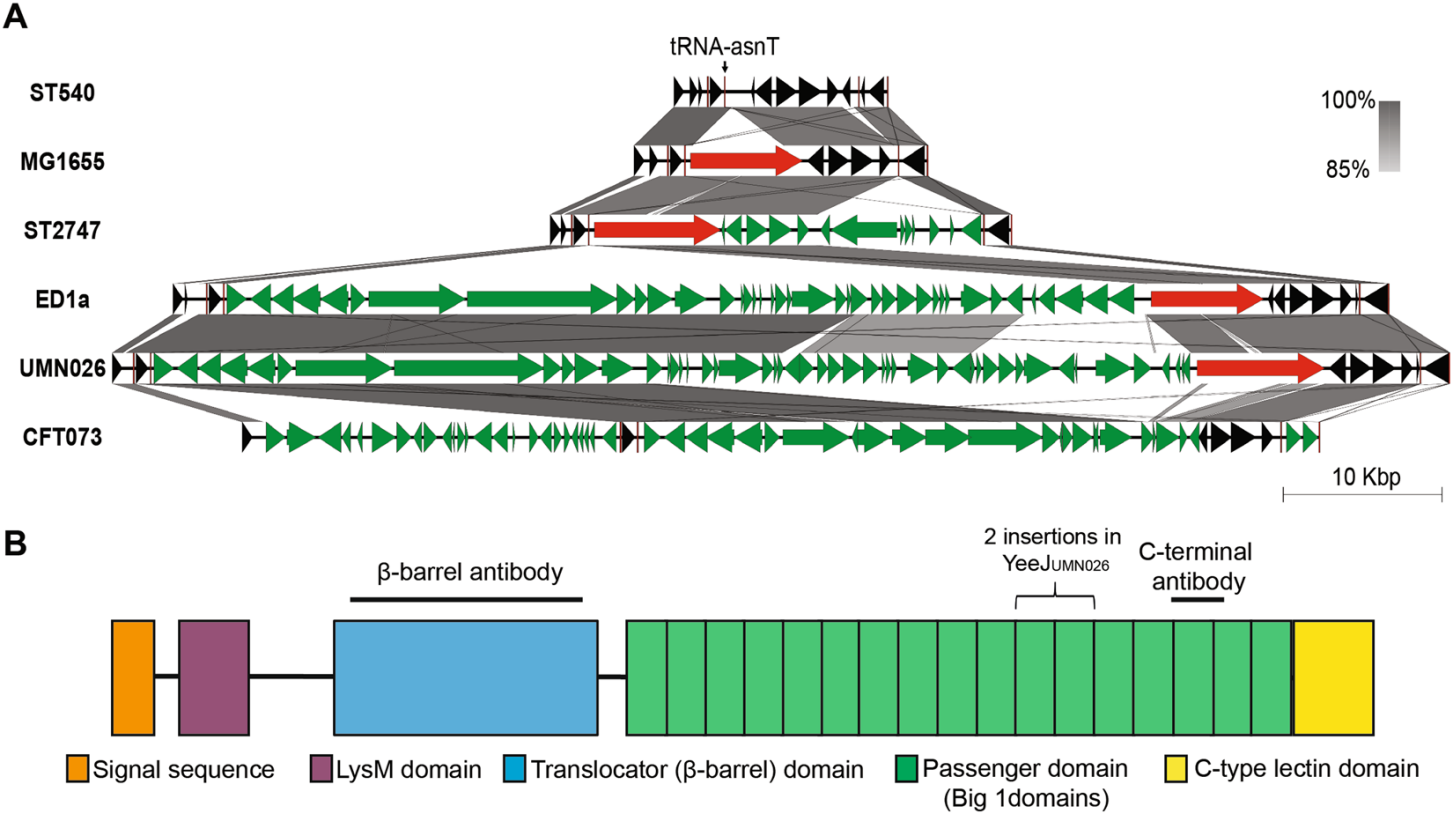
Genomic context of *yeeJ* and domain structure of the YeeJ protein. (**A**) The genomic context of *yeeJ* (red) was evaluated using MG1655 as the reference in Easyfig.^41^. Core (black arrows), variable (green arrows) and tRNA (red lines) genes are compared to the *yeeJ*-negative strains ST540 and CFT073. **(B)** Schematic illustration of the YeeJ protein from *E. coli* MG1655. Indicated are the predicted signal sequence, LysM peptidoglycan-binding domain, the translocator (β-barrel) domain, the passenger domain containing an invasin domain 3, Big 1 domains and the C-type lectin terminal domain. Also shown are the regions used to generate polyclonal antibodies against the β-barrel and passenger domains.

The prevalence of *yeeJ* was examined in a collection of 96 complete *E. coli* genome sequences representing all defined phylogroups. An intact gene encoding for a full length YeeJ protein was found in 40% (38/96) of genomes at the same genomic location as *yeeJ* in MG1655. The genetic context of *yeeJ* was conserved in the majority of strains (n = 35; represented by MG1655), while differences were observed in three strains (ST2747, ED1a and UMN026; Fig. 1A). Among the 38 *yeeJ*-positive strains, 25 belong to phylogroup A, three to B1, one to B2, two to D, six to E, and one to F (Figure S1). An additional 15 genomes contained the *yeeJ* gene at the same position, although further *in silico* analysis revealed that they had mutations or deletions that disrupt the *yeeJ* coding sequence.

Comparative analysis of the nucleotide sequence of the 38 *yeeJ* genes revealed two distinct variants, represented by *yeeJ* from MG1655 (*yeeJ*_MG1655_) and *yeeJ* from the uropathogenic strain UMN026 (*yeeJ*_UMN026_). These two variants possessed 86% nucleotide sequence conservation, with the major difference being the presence of a 906 bp fragment in *yeeJ*_UMN026_ (but absent in *yeeJ*_MG1655_). A total of 11/42 strains possessed the *yeeJ*_UMN026_ variant. Of these strains, 2/11 belonged to phylogroup A, 1/11 to B1, 0/11 to B2, 2/11 to D and 6/11 to E (Figure S1).

To extend these findings, we screened two large, well-defined *E. coli* reference collections for the presence of the *yeeJ*_MG1655_ and *yeeJ*_UMN026_ variant alleles using a two-stage PCR screening approach. These collections included the *E. coli* Reference (ECOR) collection of 72 strains^43^, as well as another previously described collection of 118 strains^42, 50–53^. Strains from both collections were isolated from an array of hosts and geographical locations, and are representative of the ecological and phylogenetic diversity of the *E. coli* species. First, the presence of *yeeJ* was examined using primers designed to amplify two conserved regions of the gene (one region corresponding to the predicted β-barrel domain and the other region corresponding to the predicted passenger domain). The correct sized product for both PCR reactions was found in 36% (26/72) of strains from the ECOR collection, and 39% (44/118) of strains from the 118-strain collection (Tables S1 and S2). Further PCR analysis of these strains using a second primer set designed to identify the 906 bp fragment revealed that the *yeeJ*_UMN026_ variant was present in 8/26 strains from the ECOR collection and 17/44 of strains from the 118-strain collection.

### Bioinformatic analysis of the YeeJ AT protein

Bioinformatic analysis of YeeJ protein from the strain MG1655 revealed a 2340 amino acid protein (instead of the 2358 amino acid protein indicated in the automated annotation). YeeJ_MG1655_ is predicted to possess a multi-domain structure: (i) an N-terminal signal sequence, (ii) a periplasmic LysM (Lysin Motif) domain (Prosite PS51782), (iii) an inverse autotransporter translocator domain (Pfam PF11924), (iv) a passenger domain containing an invasin domain 3 (Pfam PF09134) and 13 repeated bacterial immunoglobulin-like 1 domains (Pfam PF02369) and (v) a C-type lectin terminal domain (Pfam PF07979) (Fig. 1B).

The LysM domain is found in many bacterial membrane-associated and secreted proteins, and mediates direct interaction with the peptidoglycan layer to stabilize cell-surface associated proteins. It may even contribute to the formation of pores in the peptidoglycan layer that assist protein translocation^18, 54^. Modeling of the 49 amino acid YeeJ LysM domain using the PHYRE2 program^38^ indicated that it strongly resembles the domain found in other adhesins including intimin, FsaP of *Francisella tularensis* and TspA of *Neisseria meningitidis* (Figure S2)^55, 56^. The translocator domain of YeeJ revealed a predicted 12-stranded β-barrel structure (Figure S3). This domain also shares 59% amino acid sequence identity with the β-barrel domain of the intimin-like protein FdeC, and is likely to be involved in the insertion of YeeJ into the outer membrane^57^. The passenger domain is predicted to be extracellular, as described for the intimin and invasin proteins^17^ and contains 13 bacterial immunoglobulin-like domain (Big) repeats. These repeats are typical of type Ve autotransporter proteins such as intimin and FdeC^18, 57^. The last 103 amino acids of YeeJ correspond to a C-type lectin domain, a structural domain also found in intimin and some other invasins that bind to carbohydrates^58^ (Figure S4). Analysis of the YeeJ variant from strain UMN026 revealed a similar overall predicted structure, with the additional 906 bp in the passenger domain. The 906 bp fragment is predicted to encode for three additional Big domains. BLAST analysis of the 906 bp fragment revealed that it is made up of a repeated section of sequence also found in YeeJ_MG1655_.

### Cloning and expression of the *yeeJ* gene from MG1655 and UMN026

**Figure 2:**
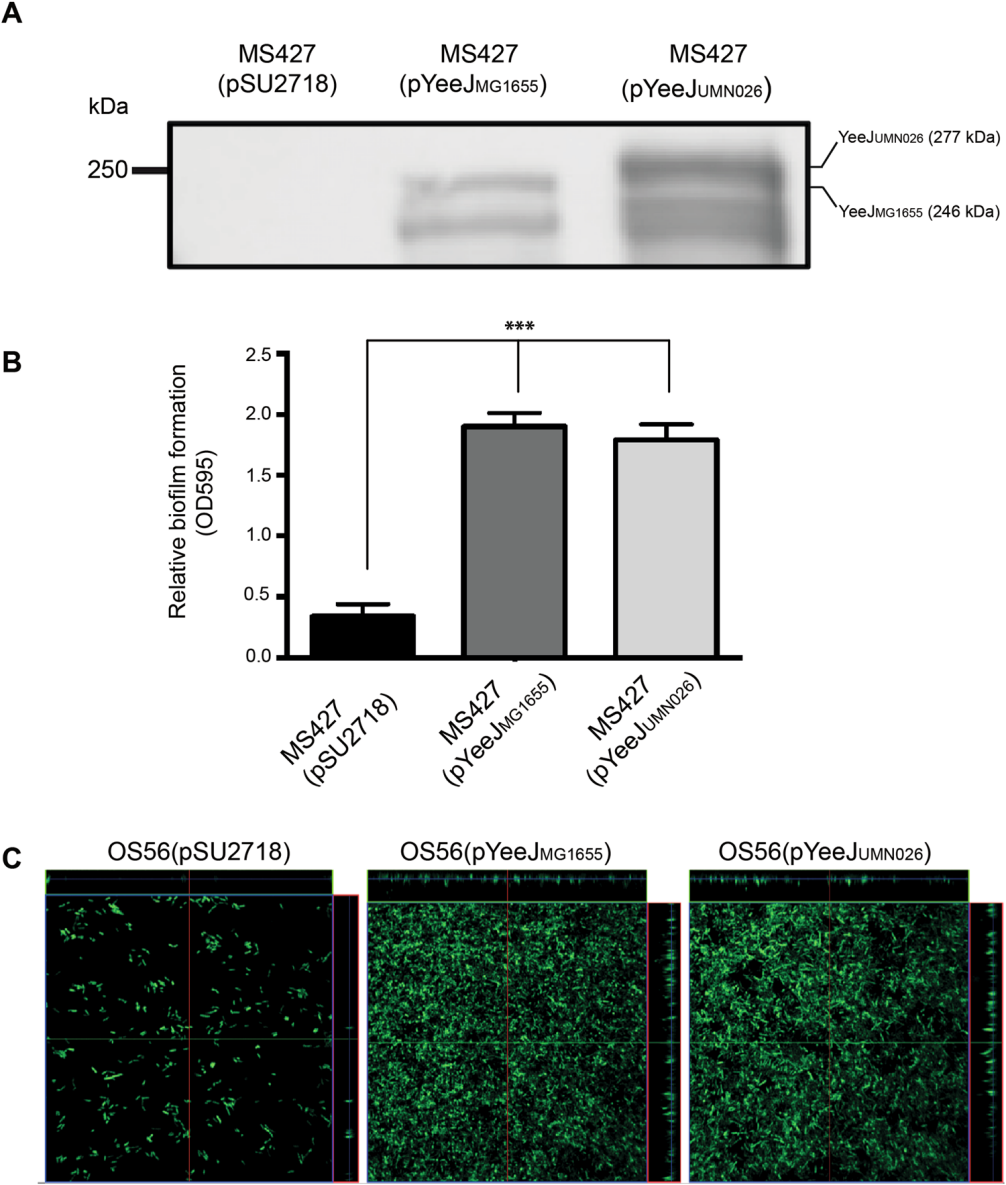
YeeJ_MG1655_ and YeeJ_UMN026_ possess similar functional properties. **(A)** Western blot analysis of whole cell lysates prepared from MS427(pSU2718) (vector control), MS427(pYeeJ_MG1655_) and MS427(pYeeJ_UMN026_). A band corresponding to full-length YeeJ from MG1655 (246KDa) and YeeJ from UMN026 (277KDa) were detected from MS427(pYeeJ_MG1655_) and MS427(pYeeJ_UMN026_), respectively. The image has been cropped from a larger blot as depicted in Supplementary Information file S7. **(B)** Biofilm formation by strains MS427 harboring plasmid pSU2718 (vector control), pYeeJ_MG1655_ or pYeeJ_UMN026_. All strains were grown in M9-Glc minimal media in the presence of 1 mM IPTG to induce *yeeJ* expression. Crystal violet staining was used to quantitate biofilm formation in 96-well microtiter plates. Values represent the average absorbance at 595 nm and error bars show the standard deviation calculated from three separate experiments. One-way ANOVA statistical analysis; ***P < 0.0001. **(C)** Scanning confocal microscopy images of biofilms formed on plastic coverslips under continuous flow conditions 24 hours post-inoculation with (i) OS56(pSU2718), (ii) OS56(pYeeJ_MG1655_) and (iii) OS56(pYeeJ_UMN026_). The images represent the horizontal sections within each biofilm. Displayed to the top and right of each micrograph are vertical sections representing the xz and yz plane, at the positions indicated by the green and red lines respectively.

In order to examine the function of YeeJ and the impact of the additional 906 bp region in *yeeJ*_UMN026_, the *yeeJ*_MG1655_ and *yeeJ*_UMN026_ genes were PCR amplified and cloned into the low copy IPTG inducible pSU2718 expression vector to generate the plasmids pYeeJ_MG1655_ and pYeeJ_UMN026_, respectively. To demonstrate the expression of the YeeJ protein, both plasmids were transformed into the *E. coli* K-12 mutant strain MS427. MS427 contains a mutation in the *flu* gene (encoding for Ag43), and is unable to form compact cell aggregates and biofilms. Western blot analysis using a YeeJ-specific antiserum raised against the YeeJ C-terminal passenger domain resulted in the detection of a band corresponding to the full-length YeeJ_MG1655_ (246 kDa) and YeeJ_UMN026_ (277 kDa) in whole cell lysates prepared from MS427(pYeeJ_MG1655_) and MS427(pYeeJ_UMN026_), respectively (Fig. 2A). Overexpression of YeeJ_MG1655_ and YeeJ_UMN026_ also resulted in multiple smaller bands presumed to be breakdown products generated during the preparation of these samples. No YeeJ-specific band was detected in whole cell lysates prepared from the MS427(pSU2718) vector control.

### Phenotypic properties of YeeJ

Some common features of AT proteins include the ability to mediate cell-to-cell aggregation, adhesion to extracellular matrix (ECM) proteins and epithelial cells, and biofilm formation. However, expression of either YeeJ_MG1655_ or YeeJ_UMN026_ in MS427 did not result in aggregation or adhesion to ECM proteins or to non-polarised and polarised Caco-2 human epithelial colorectal adenocarcinoma cells, T24 human bladder epithelial cells and MDCK dog kidney epithelial cells (data not shown). We then compared the ability of the two YeeJ variants to promote biofilm formation using two distinct systems. First, the two YeeJ proteins were tested for their ability to mediate biofilm formation in a microtiter plate biofilm assay. In this assay, MS427 (pYeeJ_MG1655_) and MS427 (pYeeJ_UMN026_) both formed a significant biofilm compared to the MS427 (pSU2718) control strain following growth in M9-Glc minimal media and induction with IPTG (Fig. 2B). Next, the two YeeJ proteins were tested for their ability to mediate biofilm formation under dynamic conditions using a continuous-flow chamber model. The *gfp*-tagged OS56 (pYeeJ_MG1655_) and OS56 (pYeeJ_UMN026_) strains were monitored for biofilm formation over 24 h using scanning confocal laser microscopy. In contrast to the OS56 (pSU2718) control strain, both OS56 (pYeeJ_MG1655_) and OS56 (pYeeJ_UMN026_) formed a biofilm with a higher total bio-volume (*P* < 0.0001), substratum coverage (*P* < 0.0001) and mean thickness (*P* < 0.0001) (Fig. 2C). No significant difference was observed between the biofilms formed by strains expressing either YeeJ variant. Taken together, these results demonstrate that YeeJ can promote biofilm formation when expressed in a recombinant *E. coli* K-12 strain, and that there is no difference between the ability of the two YeeJ variants to mediate biofilm formation under the conditions examined in these experiments.

### YeeJ is located in outer membrane vesicles and at the cell surface

**Figure 3:**
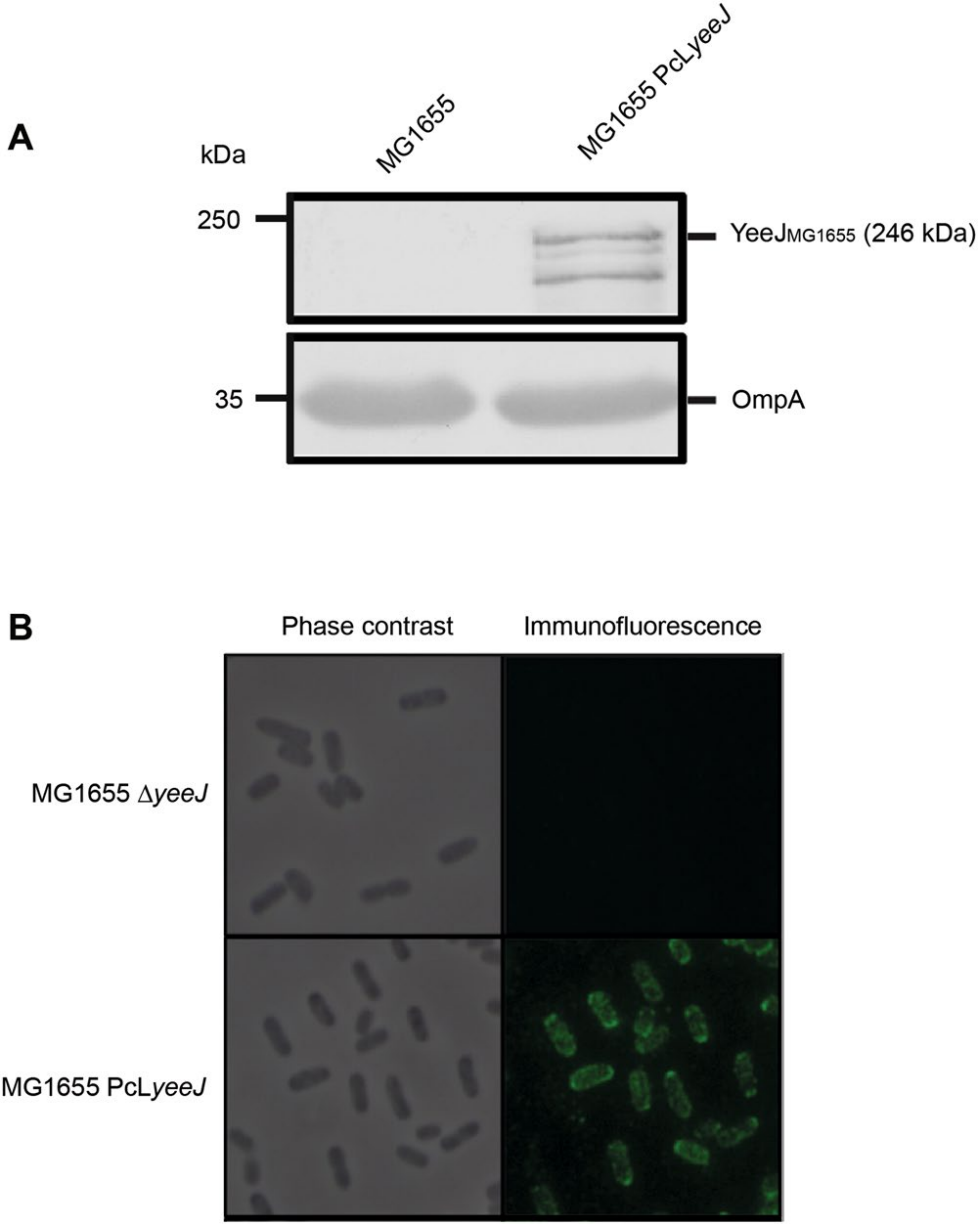
YeeJ is localized to the cell surface of *E. coli*. **(A)** Western blot analysis of outer membrane vesicles prepared from strains MG1655 and MG1655 PcL*yeeJ* using a YeeJ-specific antiserum targeting the C-terminal domain of YeeJ. A band corresponding to full-length YeeJ (246 KDa) was detected in MG1655 PcL*yeeJ* but not MG1655. The OmpA protein, which is located in the outer membrane^92^, was added as a control and was detected in both protein preparations. The images have been cropped from larger blots as depicted in Supplementary Information file S7. The same samples have been run in two independent gels, transferred and immunodetected with anti-YeeJ C-terminal or anti-OmpA antibodies. **(B)** Surface localization of the YeeJ protein. Cells of MG1655 and MG1655 PcL*yeeJ* were observed by phase contrast (left) or immunofluorescence microscopy (right) using a YeeJ-specific antiserum targeting the C-terminal domain of YeeJ.

Our bioinformatic analysis suggested that *yeeJ* encodes for a protein with structural similarities to other outer membrane proteins such as FdeC and intimin from *E. coli* ^18, 57^. As the YeeJ C-terminal passenger domain is predicted to be extracellular, we investigated the subcellular localization of YeeJ. OMVs are inherently enriched in surface exposed proteins^59^, and we previously showed that EDTA/heat-induced OMVs of uropathogenic *E. coli* are strongly enriched with OM and extracellular proteins^60, 61^. Therefore, using a strain that constitutively expresses YeeJ_MG1655_ (MG1655 PcL*yeeJ*), we first examined the presence of YeeJ in outer membrane vesicles (OMVs) by Western blot analysis using antibodies raised against the YeeJ C-terminal passenger domain. A band corresponding to the expected full-length YeeJ was detected in MG1655 PcL*yeeJ*, but not in wild type MG1655 (Fig. 3A).

In order to validate the OM localization of YeeJ and further assess whether YeeJ is exposed at the cell surface, we performed immunofluorescence microscopy on non-permeabilized cells using two anti-YeeJ antisera – (i) an antiserum against the C-terminus of the potential extracellular passenger domain and (ii) an antiserum against the outer membrane embedded β-barrel domain (Fig. 1B). While the YeeJ antiserum raised against the passenger domain reacted strongly with MG1655 PcL*yeeJ* (Fig. 3B), no signal was detected using the anti-β-barrel domain (data not shown). Taken together, our results suggest that YeeJ is localised to the outer membrane, and that the passenger domain is exposed at the cell surface.

### The mature passenger domain of YeeJ can be released from the cell surface

**Figure 4:**
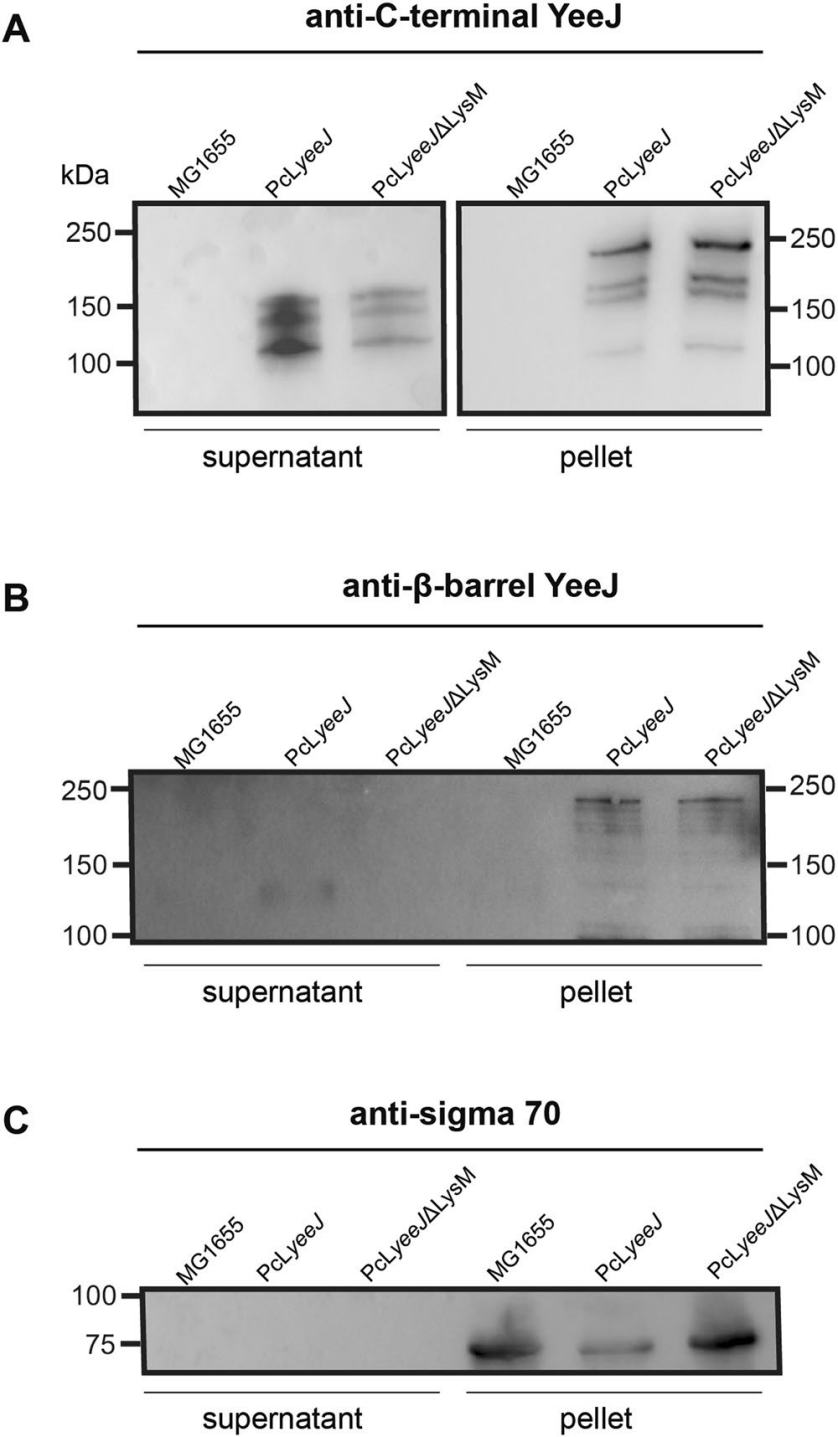
YeeJ can be cleaved from the *E. coli* cell surface. Western blot analysis of bacterial supernatants and corresponding pellets of strains MG1655, MG1655 PcL*yeeJ* and MG1655 PcL*yeeJ*ΔLysM using a polyclonal antibody targeting **(A)** the C-terminal region of YeeJ, **(B)** the β-barrel of YeeJ, and **(C)** the sigma 70 antibody (control). In (**A**) the bacterial supernatants and corresponding pellets have been run in two independent gels, transferred and immunodetected with anti-YeeJ C-terminal antibodies. In (**B**) and (**C**) the bacterial supernatants and corresponding pellets have been run in the same gel, transferred and immunodetected with YeeJ β-barrel antibodies and anti-Sigma70 antibodies, respectively. In (**A–C**) the images have been cropped from larger blots as depicted in Supplementary Information file S7.

YeeJ presence was also examined in supernatant fractions prepared from MG1655 PcL*yeeJ* (Fig. 4). Smaller bands between 125–150 kDa were detected in the TCA-precipitated supernatant using the antisera against the passenger domain. These bands were smaller than the band detected in whole cell lysates, which corresponds to the predicted full-length size of YeeJ (250 kDa) and some degradation products (Fig. 4A). Interestingly, we were not able to detect YeeJ in the supernatant fraction using the antisera against the β-barrel domain (Fig. 4B). The sigma 70 protein was used as a control, and was detected solely in the whole cell extract (pellet) and not in the supernatant (Fig. 4C). Taken together, these data suggest that the mature passenger domain of YeeJ can be processed and released into the supernatant.

### The LysM domain is involved in YeeJ binding to peptidoglycan and affects YeeJ function

Our *in silico* analysis identified a 49 amino acid LysM domain located between the signal peptide and the predicted β-barrel domain (Fig. 1B). The LysM domain confers interaction with peptidoglycan, and is present in multiple secreted proteins, outer membrane proteins, lipoproteins and proteins bound in a non-covalent manner to the bacterial cell wall^54^. We hypothesized that in YeeJ, this domain could also bind to the peptidoglycan layer of the cell and stabilize the protein. Hence, we generated an in-frame deletion of the LysM domain on the chromosome of MG1655 PcL*yeeJ*, generating the strain MG1655 PcL*yeeJ*ΔLysM. Using YeeJ-specific antisera targeting both the passenger and translocator domains we detected a band corresponding to the full length YeeJ in cell pellets of both MG1655 PcL*yeeJ* and MG1655 PcL*yeeJ*ΔLysM strains but not in MG1655 (Figure 4AB). We observed no clear differences in molecular mass of YeeJ vs YeeJ∆LysM, probably because the deletion is too small to be detected by SDS PAGE analysis.

**Figure 5:**
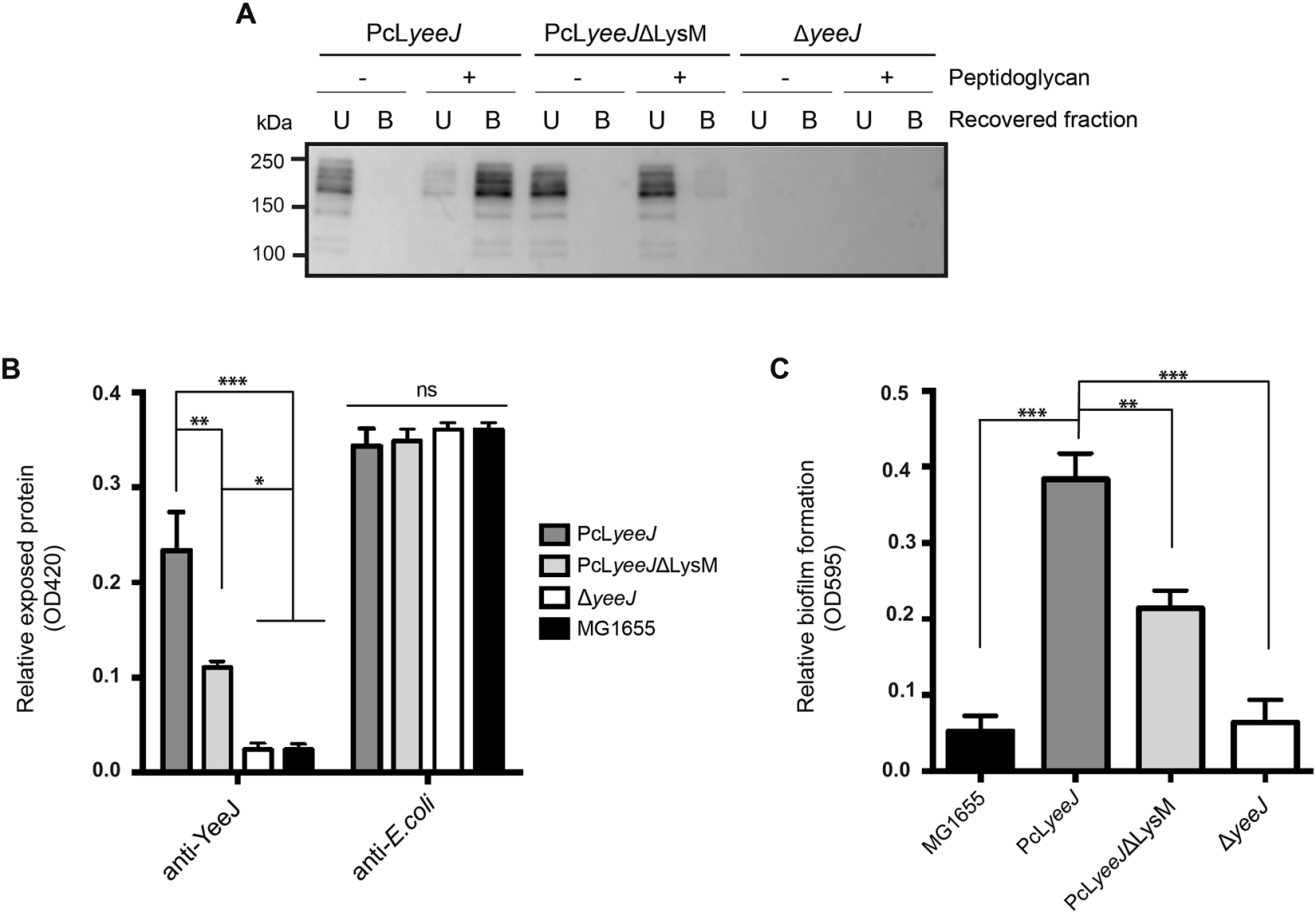
The LysM domain of YeeJ promotes binding to peptidoglycan. **(A)** Peptidoglycan pull-down assay using total protein extract from strain MG1655* ∆ yeeJ*, MG1655 PcL*yeeJ* and MG1655 PcL*yeeJ*ΔLysM obtained as described in the Materials and Methods. Total protein extracts were incubated with ultrapure peptidoglycan (Invivogen). Protein extracts containing PcL*yeeJ* with LysM motif bound to peptidoglycan (**B**), while the extracts containing PcL*yeeJ*ΔLysM were mainly associated with the unbound fraction (U). Samples were run in the same gel, transferred and immunodetected using antibodies targeted against the YeeJ passenger domain. The image corresponds to the whole blot as depicted in Supplementary Information file S7. **(B)** Quantification of YeeJ cell surface expression by strains MG1655, MG1655 Δ*yeeJ*, MG1655 PcL*yeeJ* and MG1655 PcL-*yeeJ*ΔLysM by ELISA using an antibody targeting the passenger domain of YeeJ or *E. coli* (control). One-way ANOVA statistical analysis; ***P < 0.0001; **P < 0.001; *P < 0.01. **(C)** Biofilm formation by strains MG1655, MG1655 PcL-*yeeJ*, MG1655 PcL*yeeJ*ΔLysM and MG1655 Δ*yeeJ*. All strains were grown in M9-Glc minimal medium. Crystal violet staining was used to quantitate biofilm formation in 96-well microtiter plates at 24 h. Values represent the average absorbance at 595 nm and error bars show the standard deviation calculated from three separate experiments. One-way ANOVA statistical analysis was performed using PcL*yeeJ* strain as reference: ***P < 0,0001; **P < 0,001.

Next, we carried out an *in vitro* pull-down assay, where we prepared total protein extracts from MG1655 PcL*yeeJ*, MG1655 PcL*yeeJ*ΔLysM and MG1655Δ*yeeJ* and compared their capacity to bind to commercially purified *E. coli* peptidoglycan (Fig. 5A). In these assays, we observed that the majority of YeeJ was recovered in the bound fraction (B) when using the PcL*yeeJ* protein extracts with peptidoglycan compared to the control without peptidoglycan. Conversely, the majority of YeeJΔLysM was recovered in the unbound fraction (U) when using the PcL*yeeJ*ΔLysM extracts with or without peptidoglycan. No protein was detected in either fraction prepared with MG1655Δ*yeeJ*.

We also examined whether the deletion of the LysM domain would affect YeeJ localization. We could not detect a clear difference in the cell surface localization of YeeJ between MG1655 PcL*yeeJ* and MG1655 PcL*yeeJ*ΔLysM by immunofluorescence microscopy (Figure S5). However, a more quantitative whole cell ELISA showed a reduction in signal for MG1655 PcL*yeeJ*ΔLysM compared to MG1655 PcL*yeeJ*, indicating that there was a lower amount of surface localized YeeJ in the MG1655 PcL*yeeJ*ΔLysM strain (Fig. 5B).

Finally, we performed biofilm assays to determine if the absence of the LysM domain would affect YeeJ function. In these assays, we found that despite the lack of the LysM domain, MG1655 PcL*yeeJ*ΔLysM formed a stronger biofilm compared to wild type MG1655 and MG1655Δ*yeeJ* (Fig. 5C). However, consistent with our previous data, biofilm formation by MG1655 PcL*yeeJ* was enhanced compared to MG1655 PcL*yeeJ*ΔLysM. Taken together, our results demonstrate that the LysM domain of YeeJ is able to bind to peptidoglycan, and deletion of this domain results in a reduction in the amount of YeeJ localized to the cell surface, which in turn affects biofilm formation.

### The polynucleotide phosphorylase PNP affects *yeeJ* mRNA levels

**Figure 6:**
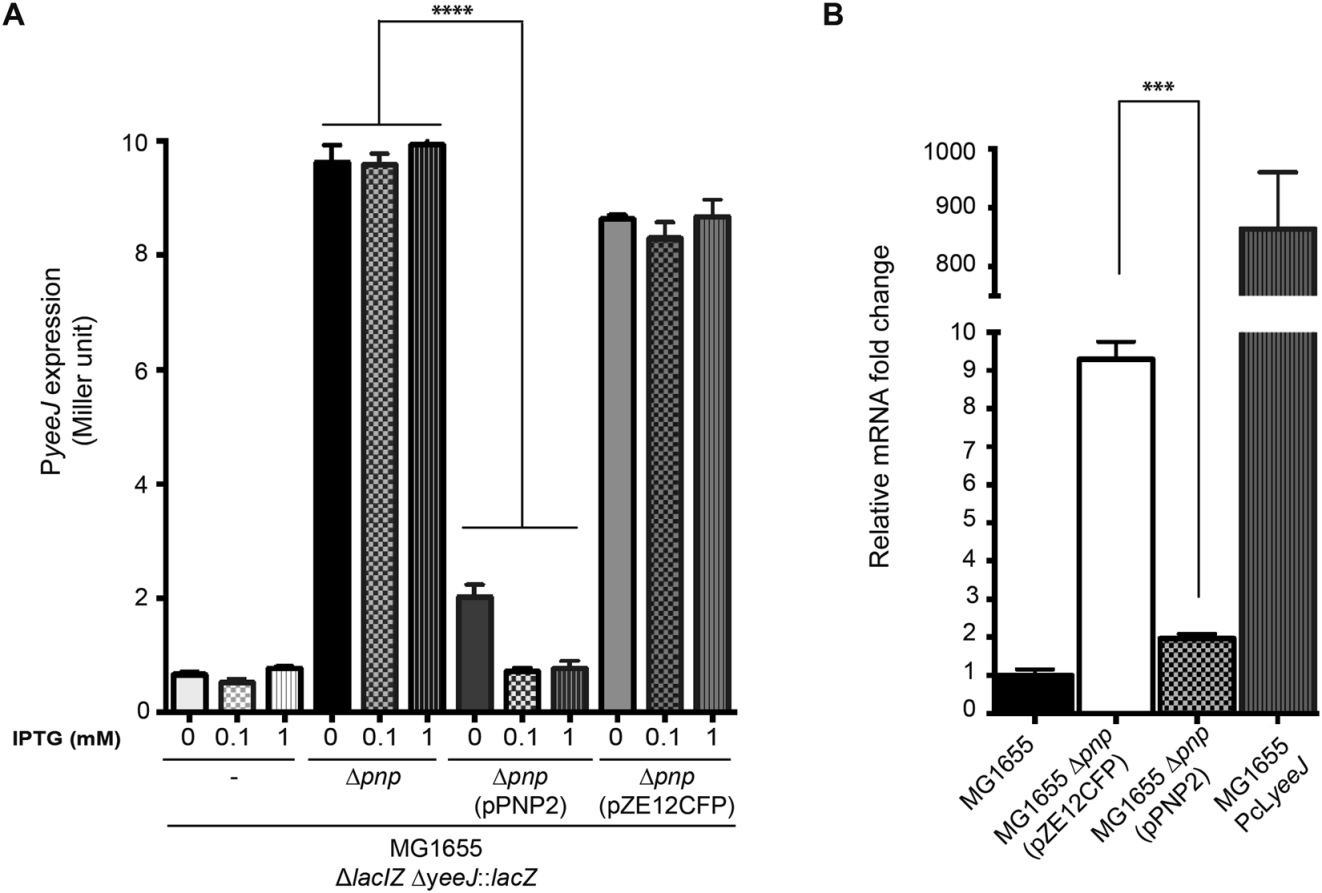
PNPase is a repressor of *yeeJ* transcription. **(A)** Activity of the *yeeJ* promoter-*lacZ* fusion assessed by β-galactosidase quantitation in wild type MG1655 (MG1655 Δ*lacIZ* Δ*yeeJ*::*lacZ*), an isogenic *pnp* mutant, complemented *pnp* mutant with plasmid pPNP2 and *pnp* mutant carrying the empty plasmid pZE12CFP. β-galactosidase levels were measured in each individual strain with different IPTG concentrations (0, 0.1 and 1 mM) from an overnight culture. Mutation of *pnp* gene increased P*yeeJ* activity and complementation of the *pnp* mutation restored the P*yeeJ* basal activity to wild type levels. All experiments were performed in triplicate. One-way ANOVA statistical analysis: ***P < 0.0001; **P < 0.001. **(B)** Transcription of the *yeeJ* gene in wild type MG1655, isogenic *pnp* mutant, complemented mutant with plasmid pPNP2 and the PcL*yeeJ* strain. The relative fold difference in *yeeJ* transcript levels relative to MG1655 as determined by qRT-PCR using the 2^−ΔΔCT^ method. Mutation of the *pnp* gene led to an increase in *yeeJ* mRNA transcript levels, and complementation of the mutant with a plasmid containing *pnp* (pPNP) restored *yeeJ* mRNA transcripts to wild type level. All experiments were performed in triplicate. One-way ANOVA statistical analysis was performed using MG1655 Δ*pnp* as reference: ***P < 0.0001.

Our western blot and ELISA analyses indicated that YeeJ is either not produced or produced at levels below our limit of detection during growth in LB broth. To investigate the genetic basis of *yeeJ* regulation, we generated a chromosomal *yeeJ* promoter-*lacZ* reporter fusion construct (MG1655 Δ*lacIZ* Δ*yeeJ*::*lacZ*). All MG1655 Δ*lacIZ* Δ*yeeJ*::*lacZ* colonies were white when grown on LB agar supplemented with X-gal at 37 °C, indicating no apparent activity from the *yeeJ* promoter, a result consistent with the lack of detection of YeeJ in MG1655 (Figs 3 and 4). Since the expression of cell surface adhesins can be modulated by environmental factors, we assessed whether *yeeJ* would be expressed in MG1655 following growth under different conditions (including different temperatures, static growth, anaerobic conditions and increased osmotic conditions). None of the tested conditions affected *yeeJ* expression (Figure S6). In order to identify potential transcriptional regulators of *yeeJ*, the MG1655 Δ*lacIZ ∆yeeJ*::*lacZ* reporter strain was subjected to random *mariner* transposon mutagenesis. The resultant transposon mutants were screened on LB plates supplemented with X-gal to identify blue colonies indicative of an active *yeeJ* promoter. Three blue transposon mutants were isolated, and the transposon insertion sites in these mutants were determined by arbitrary PCR. All three mutants contained independent insertions within the *pnp* gene (Accession Number: ECK3152). The *pnp* gene encodes for a multi-enzyme complex polynucleotide phosphorylase (PNPase), which is involved in RNA metabolism and controls numerous phenotypes such as biofilm formation, motility and bacterial survival^62–64^. In order to confirm the activity of the *yeeJ* promoter in the *pnp* mutant, we generated a specific *pnp* mutant in the MG1655 Δ*lacIZ ∆yeeJ*::*lacZ* strain and performed a ß-galactosidase assay (Fig. 6A). To complement *pnp* mutation, the *pnp* gene from strain MG1655 was amplified and cloned into the pZE12CFP plasmid, under the control of an IPTG inducible promoter, to generate plasmid pPNP2. Our results demonstrated that the activity of the *yeeJ* promoter is induced in the *pnp* mutant (MG1655 Δ*pnp* Δ*lacIZ* Δ*yeeJ*::*lacZ*), and complementation of the *pnp* mutation with pPNP2 restores promoter activity to wild type level.

To further confirm the effect of PNPase on *yeeJ* transcription, we deleted *pnp* in MG1655Δ*pnp* and we evaluated levels of these transcripts by qRT-PCR. The strain MG1655 PcL*yeeJ*, that constitutively expresses *yeeJ* was used as a positive control. The level of *yeeJ* transcript was examined in wild type MG1655, MG1655 Δ*pnp* (pZE12CFP), complemented MG1655 Δ*pnp* (pPNP2) and MG1655 PcL*yeeJ* strains by qRT-PCR (Fig. 6B). Consistent with our ß-galactosidase assay, inactivation of the *pnp* gene led to an ~9-fold increase in relative *yeeJ* transcript level, and complementation of the mutant with pPNP2 plasmid restored relative *yeeJ* transcripts to wild type level. In contrast to MG1655 Δ*pnp*, the level of *yeeJ* transcription in MG1655 PcL*yeeJ* was 900-fold higher than in MG1655. Deletion of the *pnp* gene in MG1655 did not lead to increased biofilm formation (data not shown), suggesting that a threshold level of YeeJ is required to translate into increased adhesion. Taken together, these results indicate that PNPase negatively regulates the transcription of *yeeJ*. However, this repression cannot totally explain the very low *yeeJ* expression observed during laboratory growth.

## Discussion

*E. coli* produces a vast number of factors that contribute to biofilm formation and adhesion to various surfaces, including AT protein adhesins. These adhesion factors are a core component of the type V secretion system that delivers cargo proteins across the outer membrane of Gram-negative bacteria. The Ve subclass of AT proteins are referred to as IATs, due to their similarity with classical monomeric AT proteins, but with the passenger and translocation domain in opposite locations within the primary amino acid sequence. Two well-studied proteins from this subclass include intimin and FdeC of *E. coli*, both of which have been extensively characterised^48^. Here, we characterised the YeeJ IAT protein from K-12 strain MG1655; we performed an *in silico* analysis of the *yeeJ* gene, determined the prevalence of *yeeJ*, and demonstrated that it is surface localized and mediates biofilm formation *in vitro*.

Our analysis revealed that the *yeeJ* gene in MG1655 is found immediately downstream of the tRNA-*asnT* gene, a common site for the insertion of horizontally acquired DNA^65, 66^. Indeed, the tRNA-*asnT* gene is frequently associated with insertion of the high pathogenicity island that contains genes required for the synthesis of yersiniabactin. This pathogenicity island was originally discovered in *Yersinia enterocolitica* ^67^, but has also been identified in multiple pathotypes of *E. coli* ^68–71^. Interestingly, this pathogenicity island has been shown to be more frequently associated with pathogenic *E. coli* isolates^72^. The genomic location of *yeeJ* adjacent to the tRNA-*asnT*, together with the high potential for recombination at this site, may explain why a high number of strains possessed a truncated *yeeJ* gene, and why a full length *yeeJ* gene is found more frequently in phylogroup A (non-pathogenic) strains.

Our *in silico* screen of complete *E. coli* genomes publicly available on the NCBI database revealed that 38 strains possess an intact *yeeJ* gene encoding for the full length protein. These strains belong to different pathotypes and phylogroups, suggesting that the *yeeJ* gene is conserved across a diverse range of strains. An additional 15 strains possess the *yeeJ* gene, but have frame-shift mutations that result in a truncated YeeJ protein. However, it is possible that these mutations are artifacts of sequencing or post-sequencing genome assembly errors. Indeed, this type of miss-assembly has previously been observed in the highly repetitive *upaH* AT gene from CFT073, which was initially reported to contain a frame-shift resulting in a truncated protein. Subsequent work determined that the *upaH* gene was misassembled during genome closure, and in fact it encodes for a full-length surface-expressed protein that mediates biofilm formation^73^. In addition, we also examined the presence of *yeeJ* in two large *E. coli* collections by PCR; the 72-strain ECOR collection and a collection of previously described 118 strains. Both collections correspond to strains isolated from diverse hosts and geographical sites, thus representing the ecological and phylogenetic diversity of the *E. coli* species. The correct sized PCR products were amplified in roughly 40% of strains, providing further evidence that there is strong selective pressure favouring the conservation of *yeeJ* gene in the *E. coli* species.

Although YeeJ has been previously linked to the Bap family of proteins^9, 32^, our analysis suggests that it is more closely associated with intimin and FdeC, and the IAT family of proteins. Despite having multiple Big domains (like the Bap proteins), YeeJ also contains a N-terminal LysM domain, a translocator β-barrel domain and a passenger domain capped with a C-lectin-like domain, all of which are absent in the Bap proteins but found in other IAT proteins. Moreover, the multiple repeats of Big domains (Big_3_2 (Pfam 12245) and Big_3_4 (Pfam13754)) found in Bap are different from the ones found in YeeJ. Two distinct variants of the *yeeJ* gene were identified based on the presence/absence of a 906 bp fragment within the passenger-encoding domain. Interestingly, the gene encoding for the longer variant of YeeJ was found in almost all *yeeJ*-positive pathogenic isolates identified in our bioinformatics analysis, but not in any non-pathogenic isolates. This suggests that the region encoded by this 906 bp fragment may contribute to fitness and/or virulence. Indeed, other adhesins like UpaH and Ag43 exhibit sequence variation that results in altered levels of biofilm formation by different variants^14, 74^. Hence, one of the aims of this study was to characterize both variants using the genes from *E. coli* MG1655 and UMN026 as representatives. However, both variants displayed similar phenotypic properties, suggesting that this fragment in the protein does not affect YeeJ function in the assays employed in this study. Additional Big domains found in the longer version of YeeJ might confer differential properties of YeeJ in some context that remains to be elucidated, such as extension of the protein beyond other surface structures that might otherwise mask its function.

The localization of YeeJ was investigated by immunofluorescence microscopy using two different sets of YeeJ antisera. Our results suggest that the C-terminal domain of YeeJ is exposed at the cell surface, whereas the β-barrel domain is likely embedded in the outer membrane, consistent with what has been described for intimin and invasin^75^. In agreement, immunodetection of outer membrane extractions revealed the presence of YeeJ. Analysis of supernatant fractions showed that the YeeJ passenger domain might be cleaved from the cell surface, yielding a cleavage product of approximately 100 kDa less than full-length YeeJ. Similarly, the passenger domains of other AT proteins may be processed and released into the extracellular surroundings (e.g. Pet and EspP), or cleaved but remain in contact with the cell surface via non-covalent interactions with the β-barrel domain (e.g. AIDA and Ag43)^76^. Whether the YeeJ cleavage products have a relevant function remains to be determined.

Some ATs like *E. coli* UpaG, EhaG and Ag43, or meningococcal AutA have been shown to mediate biofilm formation and cell-to-cell aggregation, resulting in the formation of bacterial clumps and flocculation^4, 6, 73, 77^. Additionally, intimin mediates adhesion of enteropathogenic *E. coli* strains to the intestinal epithelium, and invasin produced by enteropathogenic strains of *Yersinia enterocolitica* mediates binding to β1-integins^17, 78^. The intimin-like FdeC also mediates biofilm formation and colonisation of the bladder and kidney^57^. However, our results indicate that YeeJ does not mediate cell-to-cell aggregation, or adhesion to ECM proteins and different types of eukaryotic cells. We cannot exclude that the C-type lectin region of YeeJ could recognize a specific, yet unknown, receptor of some eukaryotic cells.

LysM domains are well-conserved domains found in proteins from a large variety of organisms from mammals to bacteria and viruses, and are known to bind different polysaccharides containing N-acetylglucosamine residues. Recently, the molecular mechanism behind LysM-peptidoglycan interaction was described^79^. A LysM domain is present in the N-terminal region of several adhesins including FsaP from *Francisella tularensis*, TspA from *Neisseria meningitidis* and intimin from EHEC and EPEC strains^20, 55, 80^, and the LysM domain of intimin binds to peptidoglycan^20^. Consistent with this result, we showed that YeeJ also binds to peptidoglycan, while deletion of the LysM domain from YeeJ results in loss of this interaction. Additionally, the LysM domain of intimin and other similar proteins has been shown to dimerise^20^, with interaction between the LysM domain and peptidoglycan possibly contributing to translocation. Consistent with this hypothesis, our results suggested that absence of the LysM domain of YeeJ reduced its surface localisation. It is possible that LysM-mediated interaction with peptidoglycan could stabilize YeeJ in the outer membrane. Additionally, LysM mediated dimerisation and potentially higher order oligomerisation could increase its local concentration and enhance its functional activity.

We also attempted to determine the genetic basis for the very low expression of *yeeJ* during growth under standard laboratory conditions, in order to uncover potential additional layers of its regulatory control. Using a mutagenesis approach, we identified the Polynucleotide phosphorylase (PNPase) as a potential repressor of *yeeJ*. PNPase is involved in RNA metabolism, for example it degrades various mRNAs and is involved in cold shock regulation. Loss of PNPase leads to an increase in steady-state levels of mRNA^81, 82^. It has been described that PNPase is involved in the degradation of *lac* mRNA, *rnb* mRNA and the RNA-OUT anti-sense molecule^62, 83^. The *yeeJ* mRNA can thus be added to the list of mRNA degraded by PNPase. The importance of *yeeJ* mRNA degradation in the physiology or lifestyle of *E. coli* remains to be elucidated. Since the deletion of *pnp* did not lead to increased biofilm formation, we hypothesize that the repression of *yeeJ* involves additional levels of regulatory control. The regulators and/or environmental conditions that induce *yeeJ* expression remain to be identified. Interestingly, PNPase has been described to have a pleiotropic effect on other extracellular factors. For instance, PNPase inactivation affects *E. coli* virulence, particularly increasing Tir protein content and transcription of Type III secretion system components, including intimin, Tir and EspB in *E. coli* O157:H7^84^. PNPase also negatively affects *N. meningitidis* aggregation and adhesion mediated by Type IV pili^85^, as well as transcription of the genes encoding Type IV pili and the Type III secretion system in *P. aeruginosa* ^86^. In addition to the control of *yeeJ*, it is possible that PNPase also regulates the expression of other surface factors in *E. coli*, although this remains to be demonstrated.

Taken together, our characterisation of YeeJ function and the identification of PNPase as a regulator involved in the control of its expression provides new insight into the potential role of this adhesin in *E. coli* biofilm formation.

## Electronic supplementary material

Supplementary Information
