# Supplementary material for: YeeJ is an inverse autotransporter from Escherichia coli that binds to peptidoglycan and promotes biofilm formation
Source: Sci Rep. 2017 Sep 12;7:11326. doi: 10.1038/s41598-017-10902-0 (PMC5595812; doi:10.1038/s41598-017-10902-0)
Supplement: Supplementary file 1 — Supplementary Information [file 41598_2017_10902_MOESM1_ESM.pdf]

SUPPLEMENTAL MATERIAL

YeeJ is an inverse autotransporter from *Escherichia coli* that binds to peptidoglycan and promotes biofilm formation

Marta Martinez-Gil[1£*], Kelvin G.K. Goh[2,3*], Elze Rackaityte[1%], Chizuko Sakamoto[1], Bianca Audrain[1], Danilo G. Moriel[2,3$], Makrina Totsika[2,3^], Jean-Marc Ghigo[1], Mark A. Schembri[2,3#] & Christophe Beloin[1#]

[1] Institut Pasteur, Unité de Génétique des Biofilms, 28 rue du Dr. Roux, 75724 Paris CEDEX 15, France; [2] School of Chemistry and Molecular Biosciences, The University of Queensland, Brisbane, QLD 4072, Australia; [3] Australian Infectious Diseases Research Centre, The University of Queensland, Brisbane, QLD 4072, Australia

* *These authors contributed equally to this work*

Current address: [£] Departamento de Biología Celular, Genética y Fisiología, Facultad de Ciencias. Universidad de Málaga. Málaga, Spain; [%] San Francisco, Department of Medicine, University of California, San Francisco, USA; [$] GSK Vaccines Institute for Global Health S.r.l., 53100, Siena, Italy; ^ Institute of Health and Biomedical Innovation, and School of Biomedical Sciences, Queensland University of Technology, Kelvin Grove, QLD, 4059, Australia.
[#] **To whom correspondence should be sent:** Christophe Beloin (*cbeloin@pasteur.fr*); Mark Schembri (*m.schembri@uq.edu.au*)

Running Title: The *E. coli* YeeJ inverse autotransporter adhesin

Supplemental Tables

TABLE S1: Prevalence of *yeeJ* in the 72 strains of the ECOR *E. coli* collection determined by PCR screening of two fragments of the *yeeJ* gene**.**

TABLE S2. Prevalence of *yeeJ* in a 118 strains collection of *E. coli* determined by PCR screening of two fragments of the *yeeJ* gene.

TABLE S1: Prevalence of *yeeJ* in the 72 strains of the ECOR *E. coli* collection determined by PCR screening of two fragments of the *yeeJ* gene.

Strain	Phylogroup	5' *yeeJ*	3' *yeeJ*	906bp fragment
ECOR 1	A	+	+	+
ECOR 2	A	-	-	N/A
ECOR 3	A	+	-	N/A
ECOR 4	A	+	+	-
ECOR 5	A	+	+	+
ECOR 6	A	+	+	-
ECOR 7	A	-	-	N/A
ECOR 8	A	-	-	N/A
ECOR 9	A	-	-	N/A
ECOR 10	A	-	-	N/A
ECOR 11	A	-	-	N/A
ECOR 12	A	+	+	+
ECOR 13	A	+	+	-
ECOR 14	A	+	+	+
ECOR 15	A	+	+	-
ECOR 16	A	-	-	N/A
ECOR 17	A	+	+	-
ECOR 18	A	+	-	N/A
ECOR 19	A	+	+	-
ECOR 20	A	-	-	N/A
ECOR 21	A	+	+	-
ECOR 22	A	+	+	-
ECOR 23	A	+	+	-
ECOR 24	A	-	-	N/A
ECOR 25	A	+	-	N/A
ECOR 26	B1	+	+	-
ECOR 27	B1	+	+	-
ECOR 28	B1	-	+	N/A
ECOR 29	B1	+	+	-
ECOR 30	B1	+	+	-
ECOR 31	E	+	+	+
ECOR 32	B1	+	+	-
ECOR 33	B1	+	+	-
ECOR 34	B1	+	+	-
ECOR 35	D	+	-	N/A
ECOR 36	D	+	-	N/A
ECOR 37	E	+	+	+
ECOR 38	D	+	-	N/A
ECOR 39	D	-	-	N/A
ECOR 40	D	-	-	N/A
ECOR 41	D	-	-	N/A
ECOR 42	E	+	-	N/A
ECOR 43	E	+	-	N/A
ECOR 44	D	-	-	N/A
ECOR 45	B1	+	-	N/A
ECOR 46	D	+	-	N/A
ECOR 47	D	+	+	+
ECOR 48	D	-	-	N/A
ECOR 49	D	-	-	N/A
ECOR 50	D	-	-	N/A
ECOR 51	B2	-	-	N/A
ECOR 52	B2	-	-	N/A
ECOR 53	B2	-	-	N/A
ECOR 54	B2	-	-	N/A
ECOR 55	B2	-	-	N/A
ECOR 56	B2	-	-	N/A
ECOR 57	B2	-	-	N/A
ECOR 58	B1	+	-	N/A
ECOR 59	B2	-	-	N/A
ECOR 60	B2	-	-	N/A
ECOR 61	B2	-	-	N/A
ECOR 62	B2	-	-	N/A
ECOR 63	B2	-	-	N/A
ECOR 64	B2	-	-	N/A
ECOR 65	B2	-	-	N/A
ECOR 66	B1	-	-	N/A
ECOR 67	B1	+	+	+
ECOR 68	B1	-	-	N/A
ECOR 69	B1	+	+	-
ECOR 70	B1	-	-	N/A
ECOR 71	B1	-	-	N/A
ECOR 72	B1	+	+	-

TABLE S2: Prevalence of *yeeJ* in 118-strains collection of *E. coli* determined by PCR screening of two fragments of the *yeeJ* gene.

Strain	Phylogroup	5' *yeeJ*	3' *yeeJ*	906bp fragment
431	A	+	+	-
51191	A	+	+	-
IAI16	A	+	+	-
IAI34	A	+	+	-
Ec157	A	+	+	-
Ec212	A	+	+	-
Ec193	A	+	+	-
Ec036	A	+	+	+
ROAR373	A	+	+	-
G103	A	+	+	-
Ec194	A	+	+	-
DEC12a	B1	+	+	-
TX-1	B1	+	+	-
G001	B1	+	+	-
IAI21	B1	+	+	-
Ec150	B1	+	+	-
Ec167	B1	+	+	-
Ec192	B1	+	+	-
Ec137	B1	+	+	-
Ec195	B1	+	+	-
440	B1	+	+	+
Ec111	B1	+	+	+
Ec048	B1	+	+	-
ROAR66	B1	+	+	-
Ec061	B2	+	+	-
381A	B2	+	+	-
G031	B2	+	+	-
Ec313	B2	+	+	-
Ec023	B2	+	+	+
Ec016	B2	+	+	+
Ec179	B2	+	+	+
ROAR324	C	+	+	+
G086	D	+	+	-
Ec113	D	+	+	-
Ec087	D	+	+	+
Ec029	D	+	+	+
Ec037	D	+	+	+
Ec172	D	+	+	+
Ec135	D	+	+	+
Ec330	D	+	+	+
IAI27	D	+	+	+
DEC5d	E	+	+	+
DEC3a	E	+	+	+
DEC4a	E	+	+	+
Ec044	B1	-	-	N/A
Ec051	B1	+	-	N/A
Ec057	B1	-	-	N/A
EC083	A1	-	-	N/A
Ec094	D	-	+	N/A
4 H10407	A	-	-	N/A
Ec012	A	+	-	N/A
Ec038	B1	-	-	N/A
239kh89	C	-	-	N/A
Ec014	D	+	-	N/A
1390	B1	-	-	N/A
DAEC 213	D	-	+	N/A
Ec001	B1	-	-	N/A
Ec002	A	-	-	N/A
DAEC141	D	-	-	N/A
DAEC162	F	-	-	N/A
DAEC9	F	-	-	N/A
DEC1a	B2	-	-	N/A
RS218	B2	-	-	N/A
DAEC18	B2	-	-	N/A
EC7372	B2	-	-	N/A
DAEC20	B2	-	-	N/A
C1845	B2	-	-	N/A
11097	B1	-	-	N/A
DAEC179CT	C	-	-	N/A
DEC7a	C	-	-	N/A
DAEC19	D	-	-	N/A
56390	D	+	-	N/A
17-2	A	-	-	N/A
Ec003	B2	-	-	N/A
DAEC7	A	-	-	N/A
EDL1493	A	-	-	N/A
DEC8b	B1	-	-	N/A
248/1-2	C	-	-	N/A
H-19	B1	-	-	N/A
IAI73	B2	-	-	N/A
IAI74	B2	-	-	N/A
IAI77	B2	-	-	N/A
IAI79	B2	-	-	N/A
G109	A	-	-	N/A
IAI40	A	-	+	N/A
IAI42	A	-	-	N/A
IAI44	A	-	-	N/A
IAI58	D	+	-	N/A
IAI61	B2	-	-	N/A
IAI64	B2	-	-	N/A
IAI69	B2	-	-	N/A
IAI71	B2	-	-	N/A
IAI72	B2	-	-	N/A
IAI18	A	-	-	N/A
IAI35	D	-	-	N/A
IAI36	D	-	-	N/A
IAI37	A	-	-	N/A
IAI39	D	-	-	N/A
Ec142	D	-	+	N/A
Ec152	B2	-	-	N/A
Ec183	B1	+	-	N/A
Ec102	B2	-	-	N/A
Ec109	D	+	-	N/A
Ec110	B1	+	-	N/A
Ec133	A	-	-	N/A
Ec122	B2	-	-	N/A
Ec267	B2	-	-	N/A
Ec248	D	-	-	N/A
Ec208	C	-	-	N/A
Ec206	D	-	-	N/A
ROAR372	B2	-	-	N/A
ROAR342	B2	-	-	N/A
Ec328	B3	+	-	N/A
ROAR396	D	-	-	N/A
Ec319	B1	-	+	N/A
Ec300	A	-	+	N/A
ROAR381	A	-	-	N/A
ROAR377	B2	-	-	N/A

Supplemental Figures

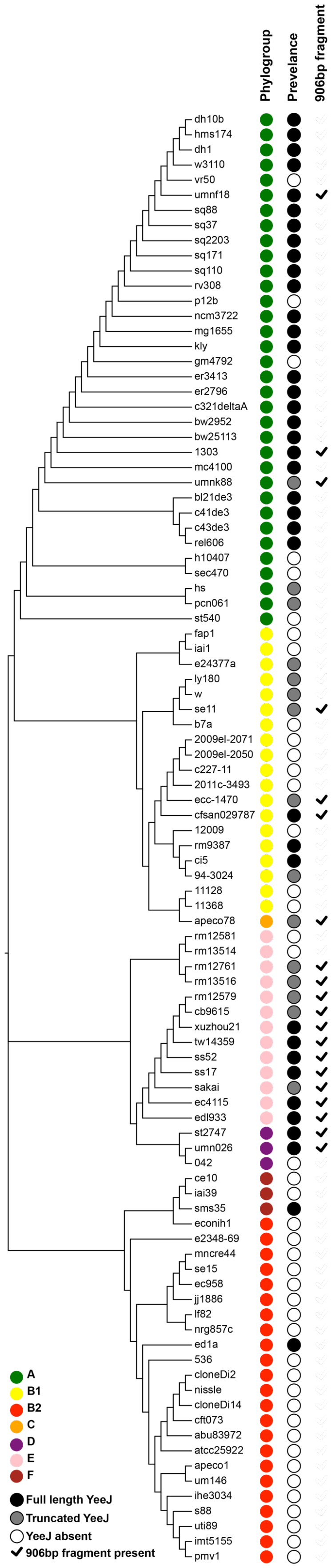

Strain	906bp fragment	Sequence type	Pathotype	Accession	Reference
dh10b		1060	Lab	CP000948	27
hms174		1060	Lab	LM993812	38
dh1		1060	Lab	CP001637	26
w3110		10	Lab	AP009048	72
vr50		10	ABU	CP011134	70
umnf18	✓	10	ETEC	AGTD01000001	68
sq88		10	Lab	CP011321	62
sq37		10	Lab	CP011320	62
sq2203		10	Lab	CP011324	62
sq171		10	Lab	CP011323	62
sq110		10	Lab	CP011322	62
rv308		10	Lab	LM995446	56
p12b		10	Lab	CP002291	50
ncm3722		10	Lab	CP011495	47
mg1655		10	Lab	CP014225	45
kly		10	Lab	CP008801	41
gm4792		10	Lab	CP011342	36
er3413		10	Lab	CP009789	34
er2796		10	Lab	CP009644	34
c321deltaA		10	Lab	CP006698	17
bw2952		10	Lab	CP001396	15
bw25113		10	Lab	CP009273	14
1303	✓	10	ExPEC	CP009166	2
mc4100		10	Lab	HG738867	44
umnk88	✓	100	ETEC	CP002729	68
bl21de3		93	Lab	CP001509	13
c41de3		93	Lab	CP010585	18
c43de3		93	Lab	CP011938	19
rel606		93	Lab	CP000819	13
h10407		48	ETEC	FN649414	37
sec470		48	EHEC	CP013962	60
hs		46	Commensal	CP000802	12
pcn061		46	ExPEC	CP006636	51
st540		540	Commensal	CP007391	65
fap1		453	?	CP009578	35
iai1		1128	Commensal	CU928160	32
e24377a		1132	ETEC	CP000800	12
ly180		1079	Lab	CP006584	43
w		1079	Lab	CP002185	71
se11	✓	156	Commensal	AP009240	58
b7a		94	ETEC	CP005998	12
2009el-2071		678	EHEC	CP003301	5
2009el-2050		678	EHEC	CP003297	5
c227-11		678	EAHEC	CP011331	16
2011c-3493		678	EAHEC	CP003289	5
ecc-1470	✓	847	ExPEC	CP010344	2
cfsan029787	✓	99	EIEC	CP011416	22
12009		17	EHEC	AP010958	3
rm9387		2773	EHEC	CP009104	6
ci5		5082	UPEC	CP011018	24
94-3024		672	EHEC	CP009106	6
11128		16	EHEC	AP010960	3
11368		21	EHEC	AP010953	3
apeco78	✓	23	APEC	CP004009	10
rm12581		32	EHEC	CP007136	54
rm13514		32	EHEC	CP006027	55
rm12761	✓	6130	EHEC	CP007133	54
rm13516	✓	6130	EHEC	CP006262	55
rm12579	✓	335	EPEC	CP003109	53
cb9615	✓	335	EPEC	CP001846	20
xuzhou21	✓	11	EHEC	CP001925	73
tw14359	✓	11	EHEC	CP001368	66
ss52	✓	11	EHEC	CP010304	64
ss17	✓	11	EHEC	CP008805	63
sakai	✓	11	EHEC	BA000007	57
ec4115	✓	11	EHEC	CP001164	29
edl933	✓	11	EHEC	CP008957	33
st2747	✓	6131	Commensal	CP007394	65
umn026	✓	597	UPEC	CU928163	32
042		414	EAEC	FN554766	4
ce10		62	NMEC	CP003034	21
iai39		62	UPEC	CU928164	32
sms35		354	Environment	CP000970	61
econih1		648	Commensal	CP009859	31
e2348-69		15	EPEC	FM180568	28
mncre44		131	ExPEC	CP010876	46
se15		131	Commensal	AP009378	59
ec958		131	UPEC	HG941718	30
jj1886		131	UPEC	CP006784	40
lf82		135	AIEC	CU651637	42
nrg857c		135	AIEC	CP001855	49
ed1a		452	Commensal	CU928162	32
536		127	UPEC	CP000247	1
cloneDi2		73	UPEC	CP002211	25
nissle		73	Probiotic	CP007799	48
cloneDi14		73	UPEC	CP002212	25
cft073		73	UPEC	AE014075	23
abu83972		73	ABU	CP001671	7
atcc25922		73	Lab	CP009072	11
apeco1		95	APEC	CP000468	9
um146		643	AIEC	CP002167	67
ihe3034		95	NMEC	CP001969	39
s88		95	NMEC	CU928161	32
uti89		95	UPEC	CP000243	69
imt5155		140	APEC	CP005930	8
pmv1		95	ExPEC	HG428755	52

Figure S1. Prevalence of *yeeJ* as determined by nucleotide BLAST in 96 complete *E. coli* genomes. Left: Phylogeny of 96 *E. coli* strains as inferred by the maximum likelihood method on the concatenated nucleotide sequence of 7 core genes used for MLST analysis. Right: Table indicating the sequence type, pathotype and accession numbers of the *E. coli* strains. The insert information depicts the symbols used to represent the different phylogroups, as well as the presence of *yeeJ* and the 906bp fragment. The figure was generated with MEGA7 [74] and Evolview [75].

1. **Hochhut B, Wilde C, Balling G, Middendorf B, Dobrindt U, Brzuszkiewicz E, Gottschalk G, Carniel E, Hacker J.** 2006. Role of pathogenicity island-associated integrases in the genome plasticity of uropathogenic *Escherichia coli* strain 536. Mol Microbiol **61:**584-595.
2. **Leimbach A, Poehlein A, Witten A, Scheutz F, Schukken Y, Daniel R, Dobrindt U.** 2015. Complete Genome Sequences of *Escherichia coli* Strains 1303 and ECC-1470 Isolated from Bovine Mastitis. Genome Announc **3**.
3. **Ogura Y, Ooka T, Iguchi A, Toh H, Asadulghani M, Oshima K, Kodama T, Abe H, Nakayama K, Kurokawa K, Tobe T, Hattori M, Hayashi T.** 2009. Comparative genomics reveal the mechanism of the parallel evolution of O157 and non-O157 enterohemorrhagic *Escherichia coli*. Proc Natl Acad Sci U S A **106:**17939-17944.
4. **Chaudhuri RR, Sebaihia M, Hobman JL, Webber MA, Leyton DL, Goldberg MD, Cunningham AF, Scott-Tucker A, Ferguson PR, Thomas CM, Frankel G, Tang CM, Dudley EG, Roberts IS, Rasko DA, Pallen MJ, Parkhill J, Nataro JP, Thomson NR, Henderson IR.** 2010. Complete genome sequence and comparative metabolic profiling of the prototypical enteroaggregative *Escherichia coli* strain 042. PLoS One **5:**e8801.

5. **Ahmed SA, Awosika J, Baldwin C, Bishop-Lilly KA, Biswas B, Broomall S, Chain PS, Chertkov O, Chokoshvili O, Coyne S, Davenport K, Detter JC, Dorman W, Erkkila TH, Folster JP, Frey KG, George M, Gleasner C, Henry M, Hill KK, Hubbard K, Insalaco J, Johnson S, Kitzmiller A, Krepps M, Lo CC, Luu T, McNew LA, Minogue T, Munk CA, Osborne B, Patel M, Reitenga KG, Rosenzweig CN, Shea A, Shen X, Strockbine N, Tarr C, Teshima H, van Gieson E, Verratti K, Wolcott M, Xie G, Sozhamannan S, Gibbons HS, Threat Characterization C.** 2012. Genomic comparison of *Escherichia coli* O104:H4 isolates from 2009 and 2011 reveals plasmid, and prophage heterogeneity, including shiga toxin encoding phage stx2. PLoS One **7:**e48228.

6. **Yan X, Fratamico PM, Bono JL, Baranzoni GM, Chen CY.** 2015. Genome sequencing and comparative genomics provides insights on the evolutionary dynamics and pathogenic potential of different H-serotypes of Shiga toxin-producing *Escherichia coli* O104. BMC Microbiol **15:**83.

7. **Zdziarski J, Brzuszkiewicz E, Wullt B, Liesegang H, Biran D, Voigt B, Gronberg-Hernandez J, Ragnarsdottir B, Hecker M, Ron EZ, Daniel R, Gottschalk G, Hacker J, Svanborg C, Dobrindt U.** 2010. Host imprints on bacterial genomes--rapid, divergent evolution in individual patients. PLoS Pathog **6:**e1001078.

8. **Zhu Ge X, Jiang J, Pan Z, Hu L, Wang S, Wang H, Leung FC, Dai J, Fan H.** 2014. Comparative genomic analysis shows that avian pathogenic *Escherichia coli* isolate IMT5155 (O2:K1:H5; ST complex 95, ST140) shares close relationship with ST95 APEC O1:K1 and human ExPEC O18:K1 strains. PLoS One **9:**e112048.

9. **Johnson TJ, Kariyawasam S, Wannemuehler Y, Mangiamele P, Johnson SJ, Doetkott C, Skyberg JA, Lynne AM, Johnson JR, Nolan LK.** 2007. The genome sequence of avian pathogenic *Escherichia coli* strain O1:K1:H7 shares strong similarities with human extraintestinal pathogenic E. coli genomes. J Bacteriol **189:**3228-3236.

10. **Mangiamele P, Nicholson B, Wannemuehler Y, Seemann T, Logue CM, Li G, Tivendale KA, Nolan LK.** 2013. Complete genome sequence of the avian pathogenic *Escherichia coli* strain APEC O78. Genome Announc **1:**e0002613.

11. **Minogue TD, Daligault HA, Davenport KW, Bishop-Lilly KA, Broomall SM, Bruce DC, Chain PS, Chertkov O, Coyne SR, Freitas T, Frey KG, Gibbons HS, Jaissle J, Redden CL, Rosenzweig CN, Xu Y, Johnson SL.** 2014. Complete Genome Assembly of *Escherichia coli* ATCC 25922, a Serotype O6 Reference Strain. Genome Announc **2**.

12. **Rasko DA, Rosovitz MJ, Myers GS, Mongodin EF, Fricke WF, Gajer P, Crabtree J, Sebaihia M, Thomson NR, Chaudhuri R, Henderson IR, Sperandio V, Ravel J.** 2008. The pangenome structure of *Escherichia coli*: comparative genomic analysis of E. coli commensal and pathogenic isolates. J Bacteriol **190:**6881-6893.

13. **Jeong H, Barbe V, Lee CH, Vallenet D, Yu DS, Choi SH, Couloux A, Lee SW, Yoon SH, Cattolico L, Hur CG, Park HS, Segurens B, Kim SC, Oh TK, Lenski RE, Studier FW, Daegelen P, Kim JF.** 2009. Genome sequences of *Escherichia coli* B strains REL606 and BL21(DE3). J Mol Biol **394:**644-652.

14. **Grenier F, Matteau D, Baby V, Rodrigue S.** 2014. Complete Genome Sequence of *Escherichia coli* BW25113. Genome Announc **2**.

15. **Ferenci T, Zhou Z, Betteridge T, Ren Y, Liu Y, Feng L, Reeves PR, Wang L.** 2009. Genomic sequencing reveals regulatory mutations and recombinational events in the widely used MC4100 lineage of *Escherichia coli* K-12. J Bacteriol **191:**4025-4029.

16. **Beaulaurier J, Zhang XS, Zhu S, Sebra R, Rosenbluh C, Deikus G, Shen N, Munera D, Waldor MK, Chess A, Blaser MJ, Schadt EE, Fang G.** 2015. Single molecule-level detection and long read-based phasing of epigenetic variations in bacterial methylomes. Nat Commun **6:**7438.

17. **Lajoie MJ, Rovner AJ, Goodman DB, Aerni HR, Haimovich AD, Kuznetsov G, Mercer JA, Wang HH, Carr PA, Mosberg JA, Rohland N, Schultz PG, Jacobson JM, Rinehart J, Church GM, Isaacs FJ.** 2013. Genomically recoded organisms expand biological functions. Science **342:**357-360.

18. **Schlegel S, Genevaux P, de Gier JW.** 2015. De-convoluting the Genetic Adaptations of E. coli C41(DE3) in Real Time Reveals How Alleviating Protein Production Stress Improves Yields. Cell Rep doi:10.1016/j.celrep.2015.02.029.

19. **Kwon SK, Kim SK, Lee DH, Kim JF.** 2015. Comparative genomics and experimental evolution of *Escherichia coli* BL21(DE3) strains reveal the landscape of toxicity escape from membrane protein overproduction. Sci Rep **5:**16076.

20. **Zhou Z, Li X, Liu B, Beutin L, Xu J, Ren Y, Feng L, Lan R, Reeves PR, Wang L.** 2010. Derivation of *Escherichia coli* O157:H7 from its O55:H7 precursor. PLoS One **5:**e8700.

21. **Lu S, Zhang X, Zhu Y, Kim KS, Yang J, Jin Q.** 2011. Complete genome sequence of the neonatal-meningitis-associated *Escherichia coli* strain CE10. J Bacteriol **193:**7005.

22. **Pettengill EA, Hoffmann M, Binet R, Roberts RJ, Payne J, Allard M, Michelacci V, Minelli F, Morabito S.** 2015. Complete Genome Sequence of Enteroinvasive *Escherichia coli* O96:H19 Associated with a Severe Foodborne Outbreak. Genome Announc **3**.

23. **Welch RA, Burland V, Plunkett G, 3rd, Redford P, Roesch P, Rasko D, Buckles EL, Liou SR, Boutin A, Hackett J, Stroud D, Mayhew GF, Rose DJ, Zhou S, Schwartz DC, Perna NT, Mobley HL, Donnenberg MS, Blattner FR.** 2002. Extensive mosaic structure revealed by the complete genome sequence of uropathogenic *Escherichia coli*. Proc Natl Acad Sci U S A **99:**17020-17024.

24. **Mehershahi KS, Abraham SN, Chen SL.** 2015. Complete Genome Sequence of Uropathogenic *Escherichia coli* Strain CI5. Genome Announc **3**.

25. **Reeves PR, Liu B, Zhou Z, Li D, Guo D, Ren Y, Clabots C, Lan R, Johnson JR, Wang L.** 2011. Rates of mutation and host transmission for an *Escherichia coli* clone over 3 years. PLoS One **6:**e26907.

26. **Suzuki S, Ono N, Furusawa C, Ying BW, Yomo T.** 2011. Comparison of sequence reads obtained from three next-generation sequencing platforms. PLoS One **6:**e19534.

27. **Durfee T, Nelson R, Baldwin S, Plunkett G, 3rd, Burland V, Mau B, Petrosino JF, Qin X, Muzny DM, Ayele M, Gibbs RA, Csorgo B, Posfai G, Weinstock GM, Blattner FR.** 2008. The complete genome sequence of *Escherichia coli* DH10B: insights into the biology of a laboratory workhorse. J Bacteriol **190:**2597-2606.

28. **Iguchi A, Thomson NR, Ogura Y, Saunders D, Ooka T, Henderson IR, Harris D, Asadulghani M, Kurokawa K, Dean P, Kenny B, Quail MA, Thurston S, Dougan G, Hayashi T, Parkhill J, Frankel G.** 2009. Complete genome sequence and comparative genome analysis of enteropathogenic *Escherichia coli* O127:H6 strain E2348/69. J Bacteriol **191:**347-354.

29. **Eppinger M, Mammel MK, Leclerc JE, Ravel J, Cebula TA.** 2011. Genomic anatomy of *Escherichia coli* O157:H7 outbreaks. Proc Natl Acad Sci U S A **108:**20142-20147.

30. **Forde BM, Ben Zakour NL, Stanton-Cook M, Phan MD, Totsika M, Peters KM, Chan KG, Schembri MA, Upton M, Beatson SA.** 2014. The complete genome sequence of *Escherichia coli* EC958: a high quality reference sequence for the globally disseminated multidrug resistant E. coli O25b:H4-ST131 clone. PLoS One **9:**e104400.

31. **Conlan S, Thomas PJ, Deming C, Park M, Lau AF, Dekker JP, Snitkin ES, Clark TA, Luong K, Song Y, Tsai YC, Boitano M, Dayal J, Brooks SY, Schmidt B, Young AC, Thomas JW, Bouffard GG, Blakesley RW, Program NCS, Mullikin JC, Korlach J, Henderson DK, Frank KM, Palmore TN, Segre JA.** 2014. Single-molecule sequencing to track plasmid diversity of hospital-associated carbapenemase-producing Enterobacteriaceae. Sci Transl Med **6:**254ra126.

32. **Touchon M, Hoede C, Tenaillon O, Barbe V, Baeriswyl S, Bidet P, Bingen E, Bonacorsi S, Bouchier C, Bouvet O, Calteau A, Chiapello H, Clermont O, Cruveiller S, Danchin A, Diard M, Dossat C, Karoui ME, Frapy E, Garry L, Ghigo JM, Gilles AM, Johnson J, Le Bouguenec C, Lescat M, Mangenot S, Martinez-Jehanne V, Matic I, Nassif X, Oztas S, Petit MA, Pichon C, Rouy Z, Ruf CS, Schneider D, Tourret J, Vacherie B, Vallenet D, Medigue C, Rocha EP, Denamur E.** 2009. Organised genome dynamics in the *Escherichia coli* species results in highly diverse adaptive paths. PLoS Genet **5:**e1000344.

33. **Latif H, Li HJ, Charusanti P, Palsson BO, Aziz RK.** 2014. A Gapless, Unambiguous Genome Sequence of the Enterohemorrhagic *Escherichia coli* O157:H7 Strain EDL933. Genome Announc **2**.

34. **Anton BP, Mongodin EF, Agrawal S, Fomenkov A, Byrd DR, Roberts RJ, Raleigh EA.** 2015. Complete Genome Sequence of ER2796, a DNA Methyltransferase-Deficient Strain of *Escherichia coli* K-12. PLoS One **10:**e0127446.

35. **de Been M, Lanza VF, de Toro M, Scharringa J, Dohmen W, Du Y, Hu J, Lei Y, Li N, Tooming-Klunderud A, Heederik DJ, Fluit AC, Bonten MJ, Willems RJ, de la Cruz F, van Schaik W.** 2014. Dissemination of cephalosporin resistance genes between *Escherichia coli* strains from farm animals and humans by specific plasmid lineages. PLoS Genet **10:**e1004776.

36. **Zhang YC, Zhang Y, Zhu BR, Zhang BW, Ni C, Zhang DY, Huang Y, Pang E, Lin K.** 2015. Genome sequences of two closely related strains of *Escherichia coli* K-12 GM4792. Stand Genomic Sci **10:**125.

37. **Crossman LC, Chaudhuri RR, Beatson SA, Wells TJ, Desvaux M, Cunningham AF, Petty NK, Mahon V, Brinkley C, Hobman JL, Savarino SJ, Turner SM, Pallen MJ, Penn CW, Parkhill J, Turner AK, Johnson TJ, Thomson NR, Smith SG, Henderson IR.** 2010. A commensal gone bad: complete genome sequence of the prototypical enterotoxigenic *Escherichia coli* strain H10407. J Bacteriol **192:**5822-5831.

38. **Mairhofer J, Krempl PM, Thallinger GG, Striedner G.** 2014. Finished Genome Sequence of *Escherichia coli* K-12 Strain HMS174 (ATCC 47011). Genome Announc **2**.

39. **Moriel DG, Bertoldi I, Spagnuolo A, Marchi S, Rosini R, Nesta B, Pastorello I, Corea VA, Torricelli G, Cartocci E, Savino S, Scarselli M, Dobrindt U, Hacker J, Tettelin H, Tallon LJ, Sullivan S, Wieler LH, Ewers C, Pickard D, Dougan G, Fontana MR, Rappuoli R, Pizza M, Serino L.** 2010. Identification of protective and broadly conserved vaccine antigens from the genome of extraintestinal pathogenic *Escherichia coli*. Proc Natl Acad Sci U S A **107:**9072-9077.

40. **Andersen PS, Stegger M, Aziz M, Contente-Cuomo T, Gibbons HS, Keim P, Sokurenko EV, Johnson JR, Price LB.** 2013. Complete Genome Sequence of the Epidemic and Highly Virulent CTX-M-15-Producing H30-Rx Subclone of *Escherichia coli* ST131. Genome Announc **1**.

41. **Fridman O, Goldberg A, Ronin I, Shoresh N, Balaban NQ.** 2014. Optimization of lag time underlies antibiotic tolerance in evolved bacterial populations. Nature **513:**418-421.

42. **Miquel S, Peyretaillade E, Claret L, de Vallee A, Dossat C, Vacherie B, Zineb el H, Segurens B, Barbe V, Sauvanet P, Neut C, Colombel JF, Medigue C, Mojica FJ, Peyret P, Bonnet R, Darfeuille-Michaud A.** 2010. Complete genome sequence of Crohn's disease-associated adherent-invasive E. coli strain LF82. PLoS One **5**.

43. **Geddes RD, Wang X, Yomano LP, Miller EN, Zheng H, Shanmugam KT, Ingram LO.** 2014. Polyamine transporters and polyamines increase furfural tolerance during xylose fermentation with ethanologenic *Escherichia coli* strain LY180. Appl Environ Microbiol **80:**5955-5964.

44. **Laehnemann D, Pena-Miller R, Rosenstiel P, Beardmore R, Jansen G, Schulenburg H.** 2014. Genomics of rapid adaptation to antibiotics: convergent evolution and scalable sequence amplification. Genome Biol Evol **6:**1287-1301.

45. **Kurylo CM, Alexander N, Dass RA, Parks MM, Altman RA, Vincent CT, Mason CE, Blanchard SC.** 2016. Genome Sequence and Analysis of *Escherichia coli* MRE600, a Colicinogenic, Nonmotile Strain that Lacks RNase I and the Type I Methyltransferase, EcoKI. Genome Biol Evol **8:**742-752.

46. **Johnson TJ, Hargreaves M, Shaw K, Snippes P, Lynfield R, Aziz M, Price LB.** 2015. Complete Genome Sequence of a Carbapenem-Resistant Extraintestinal Pathogenic *Escherichia coli* Strain Belonging to the Sequence Type 131 H30R Subclade. Genome Announc **3**.

47. **Brown SD, Jun S.** 2015. Complete Genome Sequence of *Escherichia coli* NCM3722. Genome Announc **3**.

48. **Reister M, Hoffmeier K, Krezdorn N, Rotter B, Liang C, Rund S, Dandekar T, Sonnenborn U, Oelschlaeger TA.** 2014. Complete genome sequence of the gram-negative probiotic *Escherichia coli* strain Nissle 1917. J Biotechnol **187:**106-107.

49. **Allen CA, Niesel DW, Torres AG.** 2008. The effects of low-shear stress on Adherent-invasive *Escherichia coli*. Environ Microbiol **10:**1512-1525.

50. **Liu B, Hu B, Zhou Z, Guo D, Guo X, Ding P, Feng L, Wang L.** 2012. A novel non-homologous recombination-mediated mechanism for *Escherichia coli* unilateral flagellar phase variation. Nucleic Acids Res **40:**4530-4538.

51. **Liu C, Zheng H, Yang M, Xu Z, Wang X, Wei L, Tang B, Liu F, Zhang Y, Ding Y, Tang X, Wu B, Johnson TJ, Chen H, Tan C.** 2015. Genome analysis and in vivo virulence of porcine extraintestinal pathogenic *Escherichia coli* strain PCN033. BMC Genomics **16:**717.

52. **Peris-Bondia F, Muraille E, Van Melderen L.** 2013. Complete Genome Sequence of the *Escherichia coli* PMV-1 Strain, a Model Extraintestinal Pathogenic E. coli Strain Used for Host-Pathogen Interaction Studies. Genome Announc **1**.

53. **Kyle JL, Cummings CA, Parker CT, Quinones B, Vatta P, Newton E, Huynh S, Swimley M, Degoricija L, Barker M, Fontanoz S, Nguyen K, Patel R, Fang R, Tebbs R, Petrauskene O, Furtado M, Mandrell RE.** 2012. *Escherichia coli* serotype O55:H7 diversity supports parallel acquisition of bacteriophage at Shiga toxin phage insertion sites during evolution of the O157:H7 lineage. J Bacteriol **194:**1885-1896.

54. **Cooper KK, Mandrell RE, Louie JW, Korlach J, Clark TA, Parker CT, Huynh S, Chain PS, Ahmed S, Carter MQ.** 2014. Complete Genome Sequences of Two *Escherichia coli* O145:H28 Outbreak Strains of Food Origin. Genome Announc **2**.

55. **Cooper KK, Mandrell RE, Louie JW, Korlach J, Clark TA, Parker CT, Huynh S, Chain PS, Ahmed S, Carter MQ.** 2014. Comparative genomics of enterohemorrhagic *Escherichia coli* O145:H28 demonstrates a common evolutionary lineage with *Escherichia coli* O157:H7. BMC Genomics **15:**17.

56. **Krempl PM, Mairhofer J, Striedner G, Thallinger GG.** 2014. Finished Genome Sequence of the Laboratory Strain *Escherichia coli* K-12 RV308 (ATCC 31608). Genome Announc **2**.

57. **Makino K, Yokoyama K, Kubota Y, Yutsudo CH, Kimura S, Kurokawa K, Ishii K, Hattori M, Tatsuno I, Abe H, Iida T, Yamamoto K, Onishi M, Hayashi T, Yasunaga T, Honda T, Sasakawa C, Shinagawa H.** 1999. Complete nucleotide sequence of the prophage VT2-Sakai carrying the verotoxin 2 genes of the enterohemorrhagic *Escherichia coli* O157:H7 derived from the Sakai outbreak. Genes Genet Syst **74:**227-239.

58. **Oshima K, Toh H, Ogura Y, Sasamoto H, Morita H, Park SH, Ooka T, Iyoda S, Taylor TD, Hayashi T, Itoh K, Hattori M.** 2008. Complete genome sequence and comparative analysis of the wild-type commensal *Escherichia coli* strain SE11 isolated from a healthy adult. DNA Res **15:**375-386.

59. **Toh H, Oshima K, Toyoda A, Ogura Y, Ooka T, Sasamoto H, Park SH, Iyoda S, Kurokawa K, Morita H, Itoh K, Taylor TD, Hayashi T, Hattori M.** 2010. Complete genome sequence of the wild-type commensal *Escherichia coli* strain SE15, belonging to phylogenetic group B2. J Bacteriol **192:**1165-1166.

60. **Liu H, Song L, Cai Y, Wang Y, Yu L.** 2016. Draft Genome Sequence of *Escherichia coli* Strain SEC470, Isolated from a Piglet Experiencing Diarrhea. Genome Announc **4**.

61. **Fricke WF, Wright MS, Lindell AH, Harkins DM, Baker-Austin C, Ravel J, Stepanauskas R.** 2008. Insights into the environmental resistance gene pool from the genome sequence of the multidrug-resistant environmental isolate *Escherichia coli* SMS-3-5. J Bacteriol **190:**6779-6794.

62. **Quan S, Skovgaard O, McLaughlin RE, Buurman ET, Squires CL.** 2015. Markerless *Escherichia coli* rrn Deletion Strains for Genetic Determination of Ribosomal Binding Sites. G3 (Bethesda) **5:**2555-2557.

63. **Cote R, Katani R, Moreau MR, Kudva IT, Arthur TM, DebRoy C, Mwangi MM, Albert I, Raygoza Garay JA, Li L, Brandl MT, Carter MQ, Kapur V.** 2015.

Comparative analysis of super-shedder strains of *Escherichia coli* O157:H7 reveals distinctive genomic features and a strongly aggregative adherent phenotype on bovine rectoanal junction squamous epithelial cells. PLoS One **10:**e0116743.

64. **Katani R, Cote R, Raygoza Garay JA, Li L, Arthur TM, DebRoy C, Mwangi MM, Kapur V.** 2015. Complete Genome Sequence of SS52, a Strain of *Escherichia coli* O157:H7 Recovered from Supershedder Cattle. Genome Announc **3**.

65. **Xavier BB, Vervoort J, Stewardson A, Adriaenssens N, Coenen S, Harbarth S, Goossens H, Malhotra-Kumar S.** 2014. Complete Genome Sequences of Nitrofurantoin-Sensitive and -Resistant *Escherichia coli* ST540 and ST2747 Strains. Genome Announc **2**.

66. **Kulasekara BR, Jacobs M, Zhou Y, Wu Z, Sims E, Saenphimmachak C, Rohmer L, Ritchie JM, Radey M, McKevitt M, Freeman TL, Hayden H, Haugen E, Gillett W, Fong C, Chang J, Beskhlebnaya V, Waldor MK, Samadpour M, Whittam TS, Kaul R, Brittnacher M, Miller SI.** 2009. Analysis of the genome of the *Escherichia coli* O157:H7 2006 spinach-associated outbreak isolate indicates candidate genes that may enhance virulence. Infect Immun **77:**3713-3721.

67. **Krause DO, Little AC, Dowd SE, Bernstein CN.** 2011. Complete genome sequence of adherent invasive *Escherichia coli* UM146 isolated from Ileal Crohn's disease biopsy tissue. J Bacteriol **193:**583.

68. **Shepard SM, Danzeisen JL, Isaacson RE, Seemann T, Achtman M, Johnson TJ.** 2012. Genome sequences and phylogenetic analysis of K88- and F18-positive porcine enterotoxigenic *Escherichia coli*. J Bacteriol **194:**395-405.

69. **Chen SL, Hung CS, Xu J, Reigstad CS, Magrini V, Sabo A, Blasiar D, Bieri T, Meyer RR, Ozersky P, Armstrong JR, Fulton RS, Latreille JP, Spieth J, Hooton TM, Mardis ER, Hultgren SJ, Gordon JI.** 2006. Identification of genes subject to

positive selection in uropathogenic strains of *Escherichia coli*: a comparative genomics approach. Proc Natl Acad Sci U S A **103:**5977-5982.

70. **Beatson SA, Ben Zakour NL, Totsika M, Forde BM, Watts RE, Mabbett AN, Szubert JM, Sarkar S, Phan MD, Peters KM, Petty NK, Alikhan NF, Sullivan MJ, Gawthorne JA, Stanton-Cook M, Nhu NT, Chong TM, Yin WF, Chan KG, Hancock V, Ussery DW, Ulett GC, Schembri MA.** 2015. Molecular analysis of asymptomatic bacteriuria *Escherichia coli* strain VR50 reveals adaptation to the urinary tract by gene acquisition. Infect Immun **83:**1749-1764.

71. **Archer CT, Kim JF, Jeong H, Park JH, Vickers CE, Lee SY, Nielsen LK.** 2011. The genome sequence of E. coli W (ATCC 9637): comparative genome analysis and an improved genome-scale reconstruction of E. coli. BMC Genomics **12:**9.

72. **Hayashi K, Morooka N, Yamamoto Y, Fujita K, Isono K, Choi S, Ohtsubo E, Baba T, Wanner BL, Mori H, Horiuchi T.** 2006. Highly accurate genome sequences of *Escherichia coli* K-12 strains MG1655 and W3110. Mol Syst Biol **2:**2006 0007.

73. **Xiong Y, Wang P, Lan R, Ye C, Wang H, Ren J, Jing H, Wang Y, Zhou Z, Bai X, Cui Z, Luo X, Zhao A, Wang Y, Zhang S, Sun H, Wang L, Xu J.** 2012. A novel *Escherichia coli* O157:H7 clone causing a major hemolytic uremic syndrome outbreak in China. PLoS One **7:**e36144.

74. **Kumar S, Stecher G, Tamura K.** 2016. MEGA7: Molecular Evolutionary Genetics Analysis Version 7.0 for Bigger Datasets. Mol Biol Evol **33:**1870-1874.

75. **He Z, Zhang H, Gao S, Lercher MJ, Chen WH, Hu S.** 2016. Evolview v2: an online visualization and management tool for customized and annotated phylogenetic trees. Nucleic Acids Res **44:**W236-241.

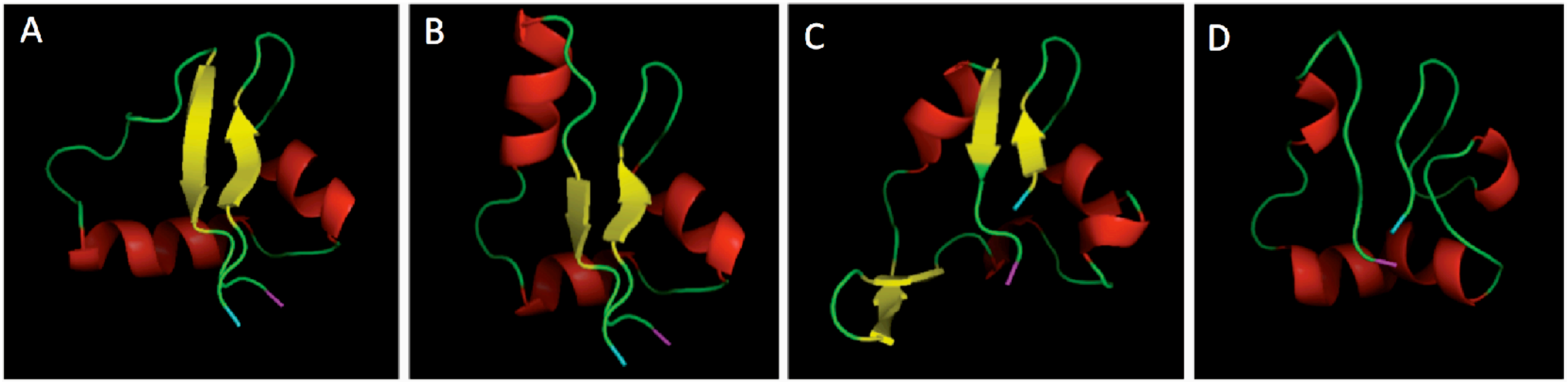

Figure S2. Models of the LysM domains in adhesins. The structure of the four LysM domains of respectively the Intimin EaeA (A), YeeJ (B), FsaP(C) and TspA (D) was modelled using the PHYRE2 program with the reference of the structure of the LysM domain of the MltD protein from *E. coli*. The N-terminal residues of the four proteins are in blue while their C-terminal residues are in magenta.

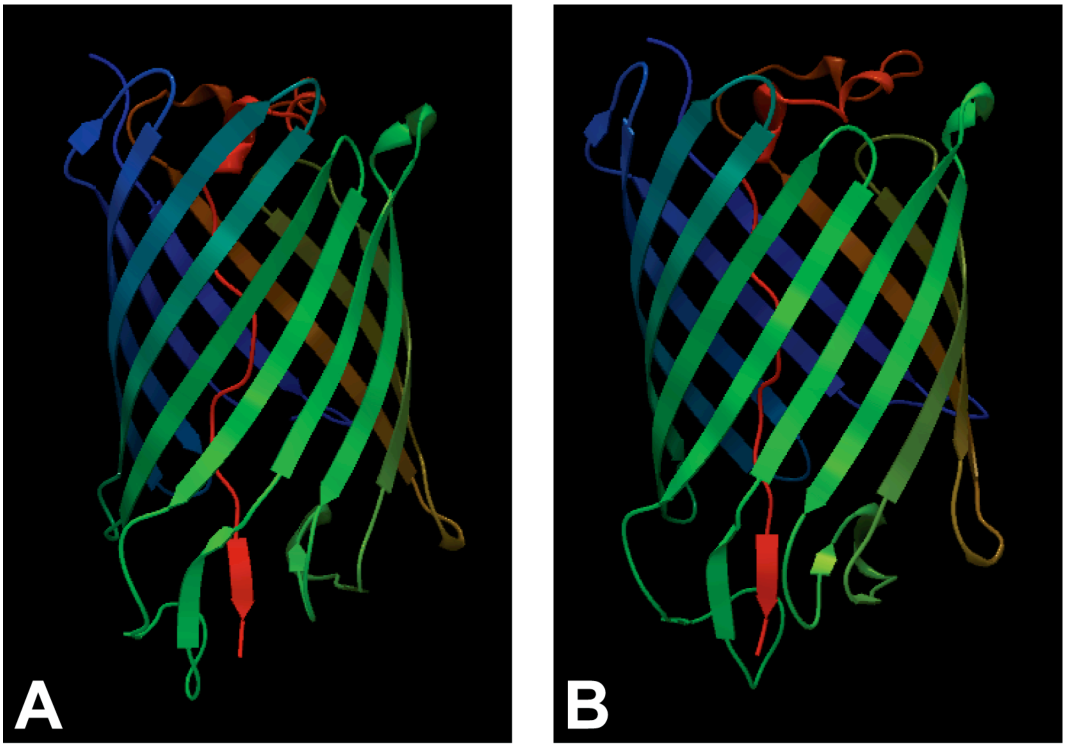

Figure S3. Model structure of the ß-barrel domain involved in the insertion of (A) Intimin and (B) YeeJ in the outer membrane. The program PHYRE2 was used to generate these models based on the structure of the ß-barrel domain of the invasin protein from *Yersinia pseudotuberculosis*.

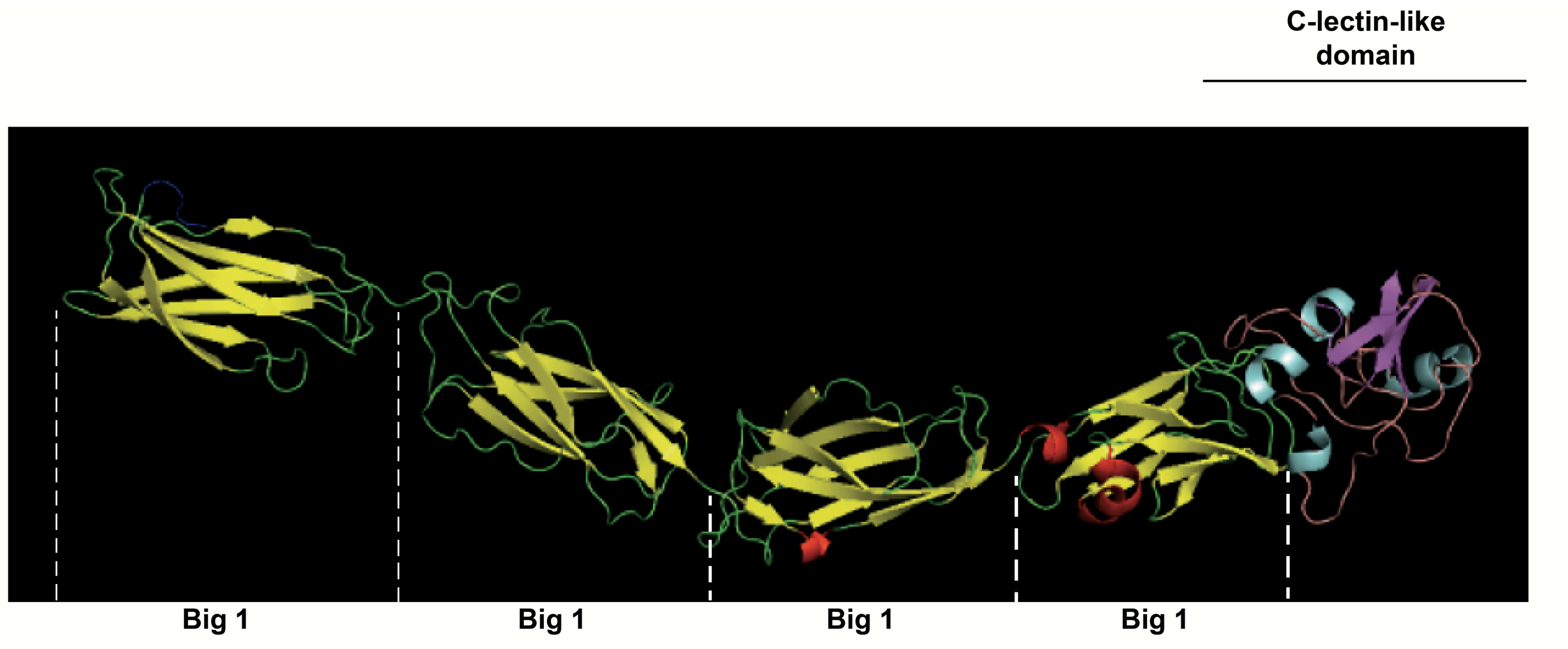

Figure S4. Structural modeling of the last 504 aa of YeeJ. Potential structure of the last 504 aa YeeJ generated by the program PHYRE2. The Big-1 and C-lectin-like domain are indicated.

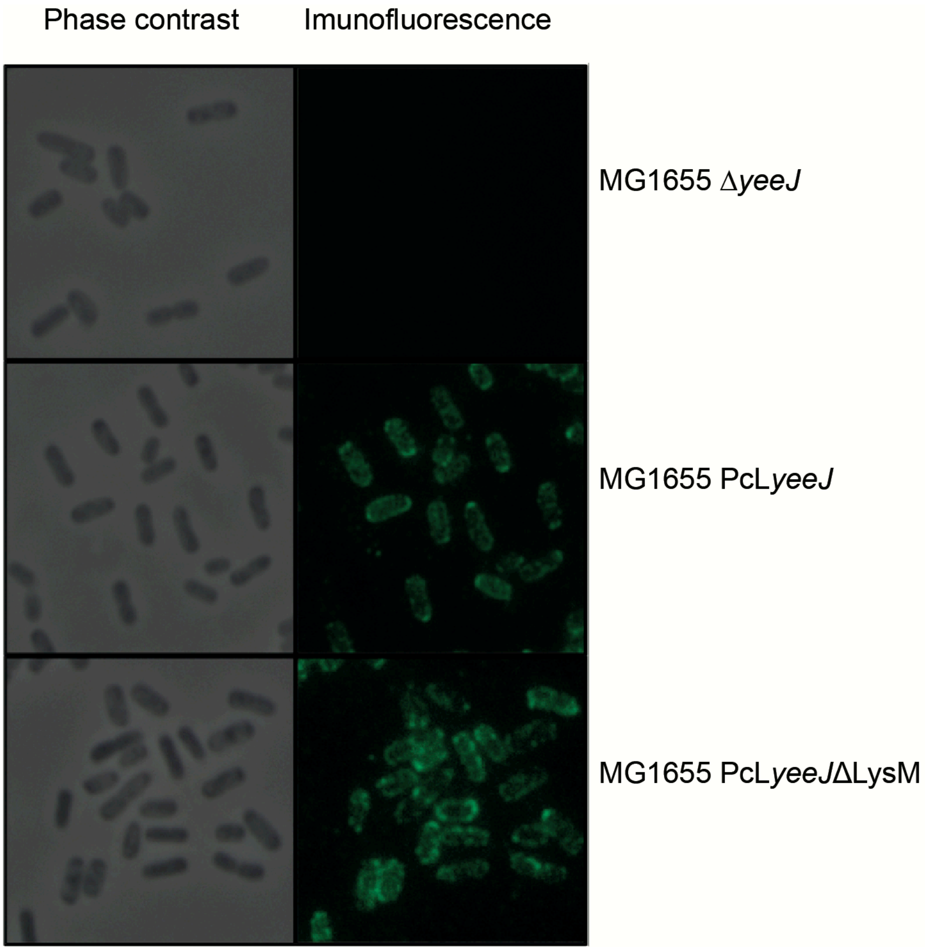

Figure S5. Effect of the LysM domain on YeeJ surface localization as evaluated using immunofluorescence. Cells of the strains MG1655 Δ*yeeJ,* MG1655 PcL*yeeJ* and MG1655 PcL*yeeJ*ΔlysM were observed by phase contrast (left) or immunofluorescence microscopy (right) using the C-terminal domain antibody against YeeJ.

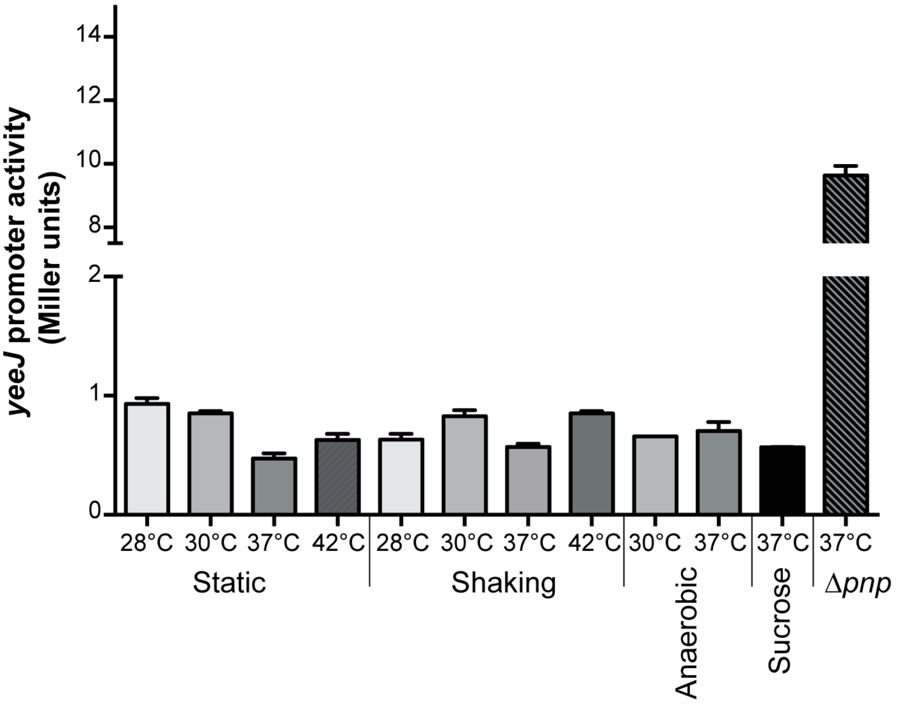

Figure S6. **Effect of environmental conditions on *yeeJ* promoter activity.** The MG1655 Δ*lacIZ* Δ*yeeJ*::*lacZ* reporter strain was grown overnight in LB broth under different conditions: 28°C, 30°C, 37°C and 42°C under both static and shaking conditions; 30°C and 37°C under anaerobic conditions; and at 37°C in the presence of 20% sucrose. The MG1655 Δ*pnp* Δ*lacIZ* Δ*yeeJ*::*lacZ* strain (last bar; far right) was used as a positive control for *yeeJ* promoter activity. All experiments were performed in triplicate.

Supplementary Information file S7

Western Blot used for Figure 2A

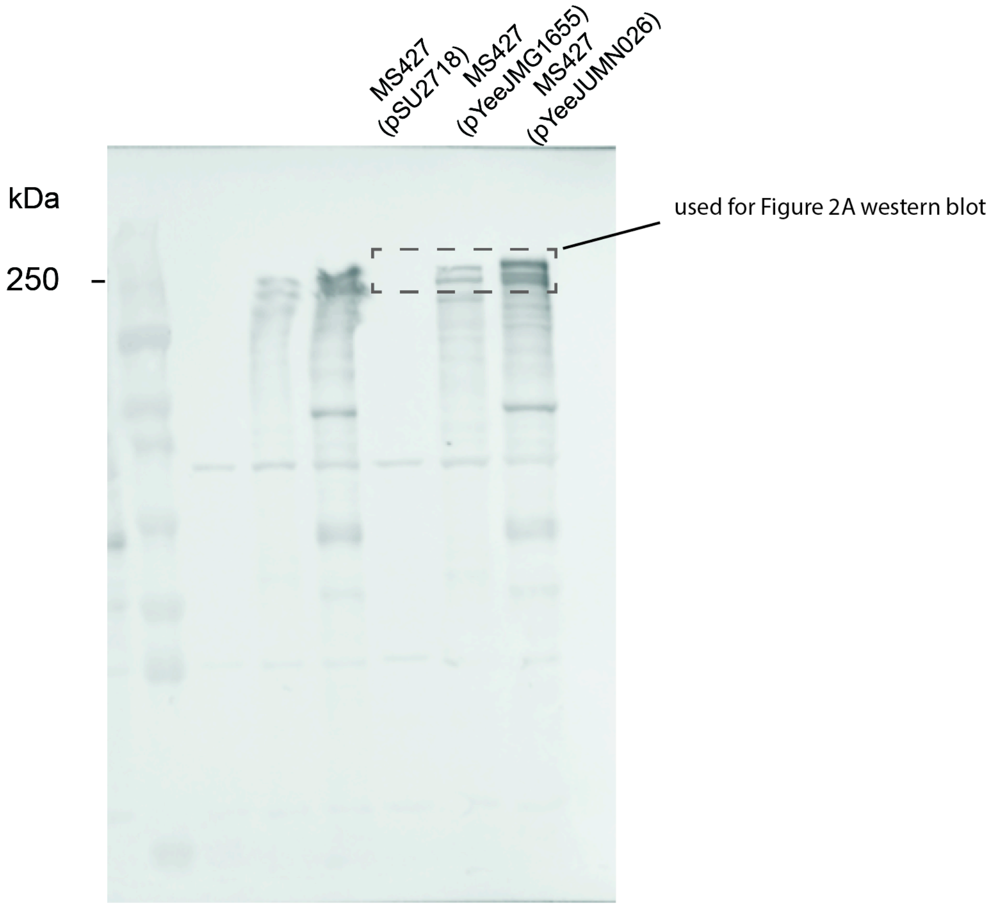

YeeJ blot
with antibodies raised against the YeeJ Cterminal passenger domain

The samples have been run in on gel, transferred
and immunodetected with anti-YeeJ antibodies.
The cropped area used to construct Figure 2A western is indicated by a grey dotted box

Western Blots used for Figure 3A

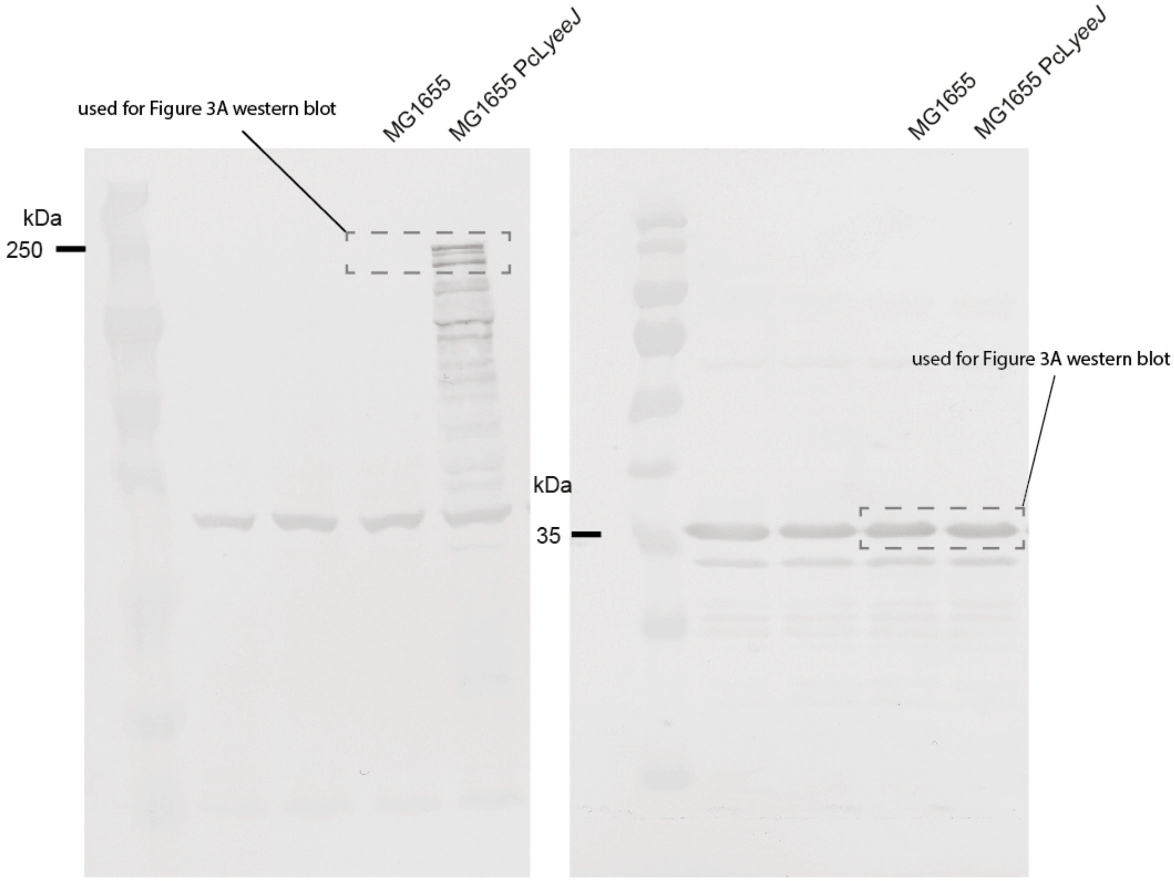

YeeJ blot
with antibodies raised against the YeeJ

OmpA blot

The same samples have been run in two independent gels, transferred
and immunodetected with anti-YeeJ (left) or anti-OmpA antibodies (right).
The cropped area used to construct Figure 3A western are indicated by grey dotted boxes

Western Blots used for Figure 4A

Left Panel
(supernatant)

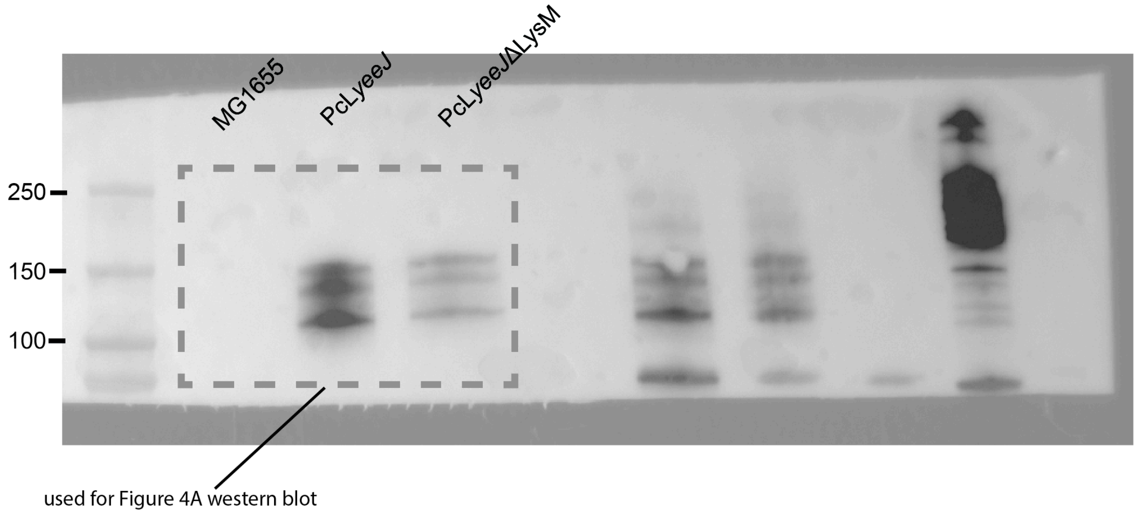

YeeJ blot
with antibodies raised against the YeeJ Cterminal passenger domain

Right Panel
(Pellets)

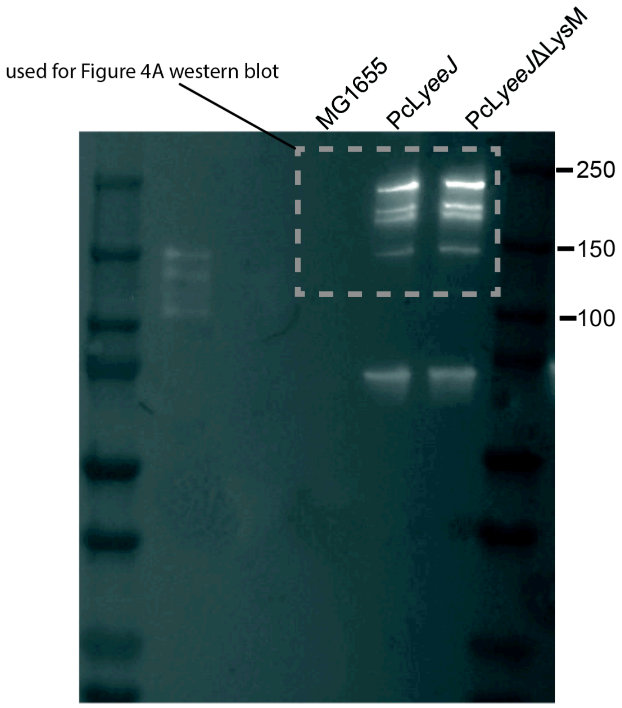

YeeJ blot
with antibodies raised against the YeeJ Cterminal passenger domain

The same bacterial supernatants and corresponding pellets samples used in Figure 4(B) and 4(C) have been run in two independent gels, transferred and immunodetected with antibodies raised against the YeeJ Cterminal passenger domain.
The cropped area used to construct Figure 4A western are indicated by grey dotted boxes

Western Blots used for Figure 4B

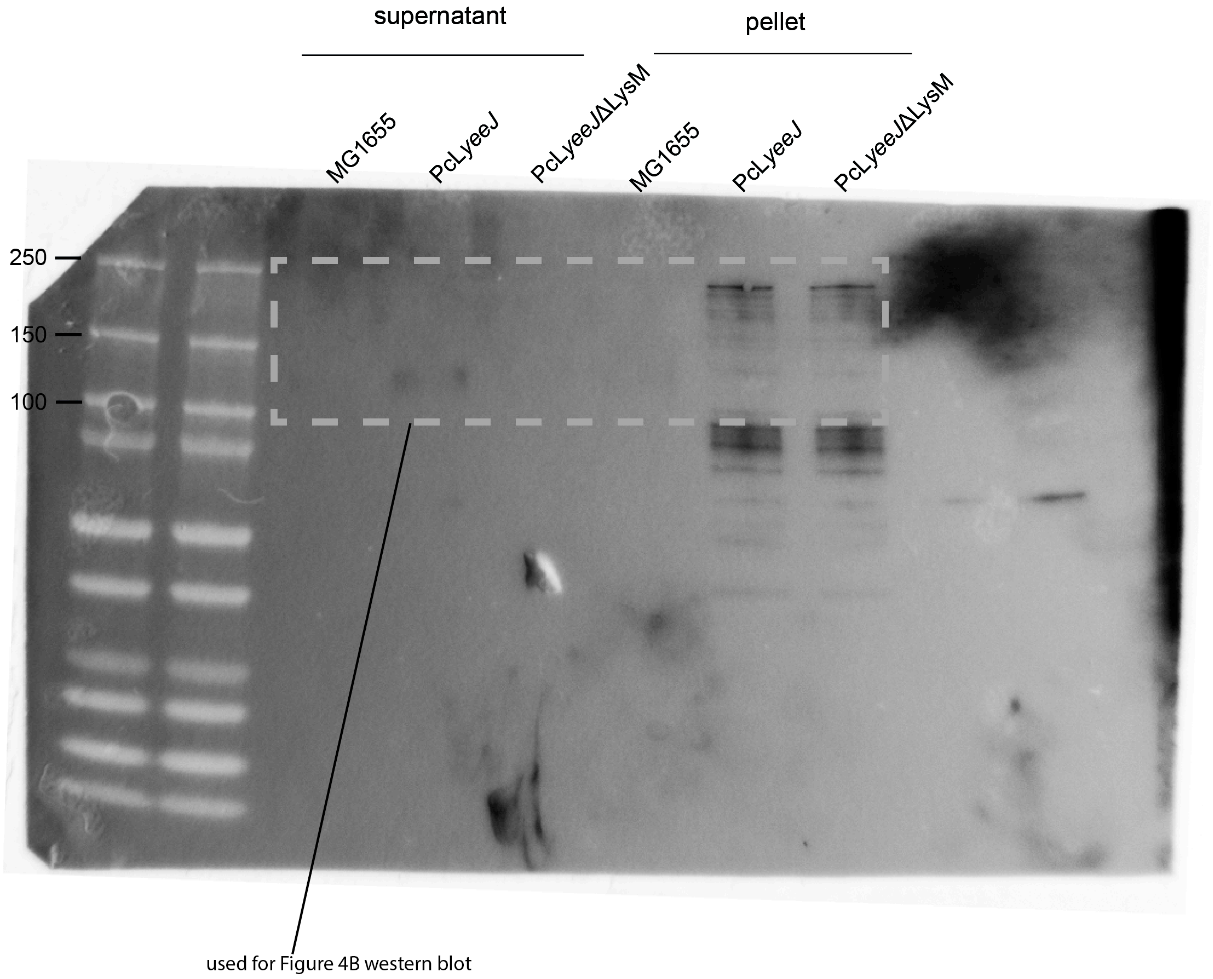

YeeJ blot
with antibodies raised against the YeeJ Betabarrel domain

The same bacterial supernatants and corresponding pellets samples used in Figure 4(A) and 4(C) have been run in the same gel, transferred and immunodetected with antibodies raised against the YeeJ Betabarrel domain domain.
The cropped area used to construct Figure 4B western isindicated by grey dotted box

Western Blots used for Figure 4C

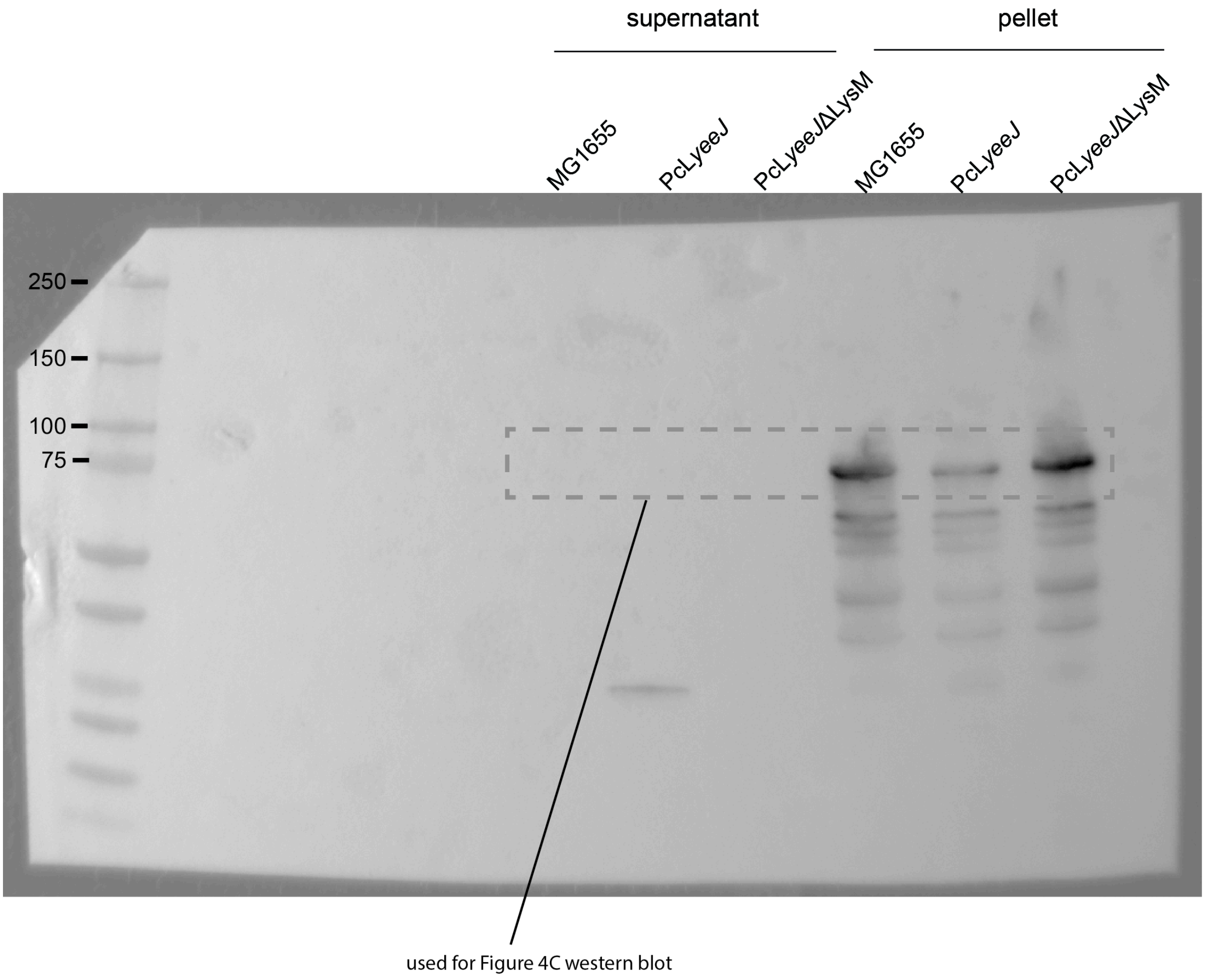

Sigma 70 blot
with antibodies raised against Sigma 70

The same bacterial supernatants and corresponding pellets samples used in Figure 4(A) and 4(B) have been run in one gel, transferred and immunodetected with antibodies raised against Sigma 70.
The cropped area used to construct Figure 4C western is indicated by a grey dotted box

Western Blots used for Figure 5A

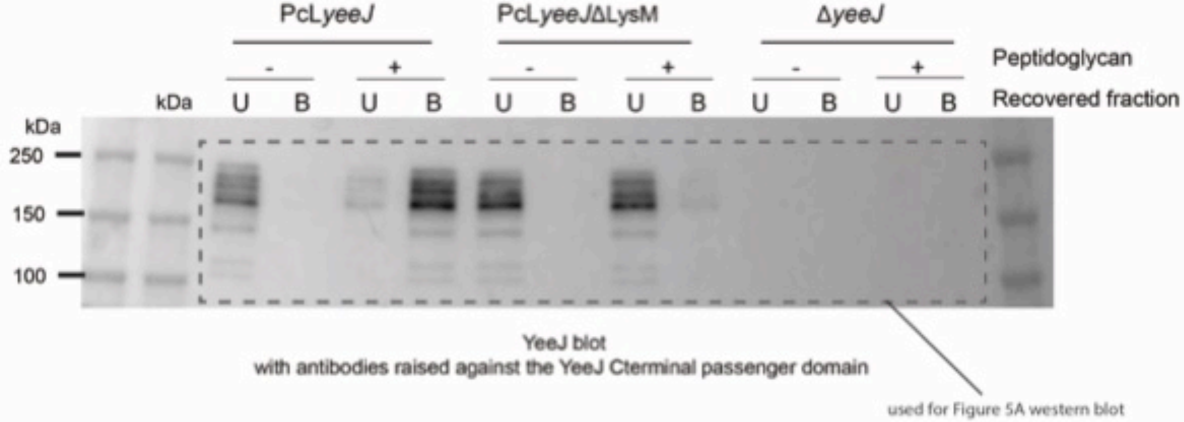

YeeJ blot
with antibodies raised against the YeeJ Cterminal passenger domain

The samples have been run in on gel, transferred
and immunodetected with anti-YeeJ antibodies.
The cropped area used to construct Figure 5A western is indicated by a grey dotted box
